# Targeting oxidative stress as a preventive and therapeutic approach for cardiovascular disease

**DOI:** 10.1186/s12967-023-04361-7

**Published:** 2023-08-02

**Authors:** Qian Yan, Shasha Liu, Yang Sun, Chen Chen, Songwei Yang, Meiyu Lin, Junpeng Long, Jiao Yao, Yuting Lin, Fan Yi, Lei Meng, Yong Tan, Qidi Ai, Naihong Chen, Yantao Yang

**Affiliations:** 1 Hunan Engineering Technology Center of Standardization and Function of Chinese Herbal Decoction Pieces, College of Pharmacy, Hunan University of Chinese Medicine, Changsha, 410208 China; 2 Department of Pharmacy, Changsha Hospital for Matemal&Child Health Care, Changsha, People’s Republic of China; 3 Department of Pharmacy, The First Hospital of Lanzhou University, Lanzhou, 730000 China; 4 Key Laboratory of Cosmetic, China National Light Industry, Beijing Technology and Business University, Beijing, 100048 China; 5 Department of Nephrology, Xiangtan Central Hospital, Xiangtan, 411100 China; 6 State Key Laboratory of Bioactive Substances and Functions of Natural Medicines, Institute of Materia Medica & Neuroscience Center, Chinese Academy of Medical Sciences and Peking Union Medical College, Beijing, 100050 China

**Keywords:** Cardiovascular disease, Oxidative stress, Herb monomer, Antioxidative

## Abstract

Cardiovascular diseases (CVDs) continue to exert a significant impact on global mortality rates, encompassing conditions like pulmonary arterial hypertension (PAH), atherosclerosis (AS), and myocardial infarction (MI). Oxidative stress (OS) plays a crucial role in the pathogenesis and advancement of CVDs, highlighting its significance as a contributing factor. Maintaining an equilibrium between reactive oxygen species (ROS) and antioxidant systems not only aids in mitigating oxidative stress but also confers protective benefits on cardiac health. Herbal monomers can inhibit OS in CVDs by activating multiple signaling pathways, such as increasing the activity of endogenous antioxidant systems and decreasing the level of ROS expression. Given the actions of herbal monomers to significantly protect the normal function of the heart and reduce the damage caused by OS to the organism. Hence, it is imperative to recognize the significance of herbal monomers as prospective therapeutic interventions for mitigating oxidative damage in CVDs. This paper aims to comprehensively review the origins and mechanisms underlying OS, elucidate the intricate association between CVDs and OS, and explore the therapeutic potential of antioxidant treatment utilizing herbal monomers. Furthermore, particular emphasis will be placed on examining the cardioprotective effects of herbal monomers by evaluating their impact on cardiac signaling pathways subsequent to treatment.

**Graphical Abstract:**

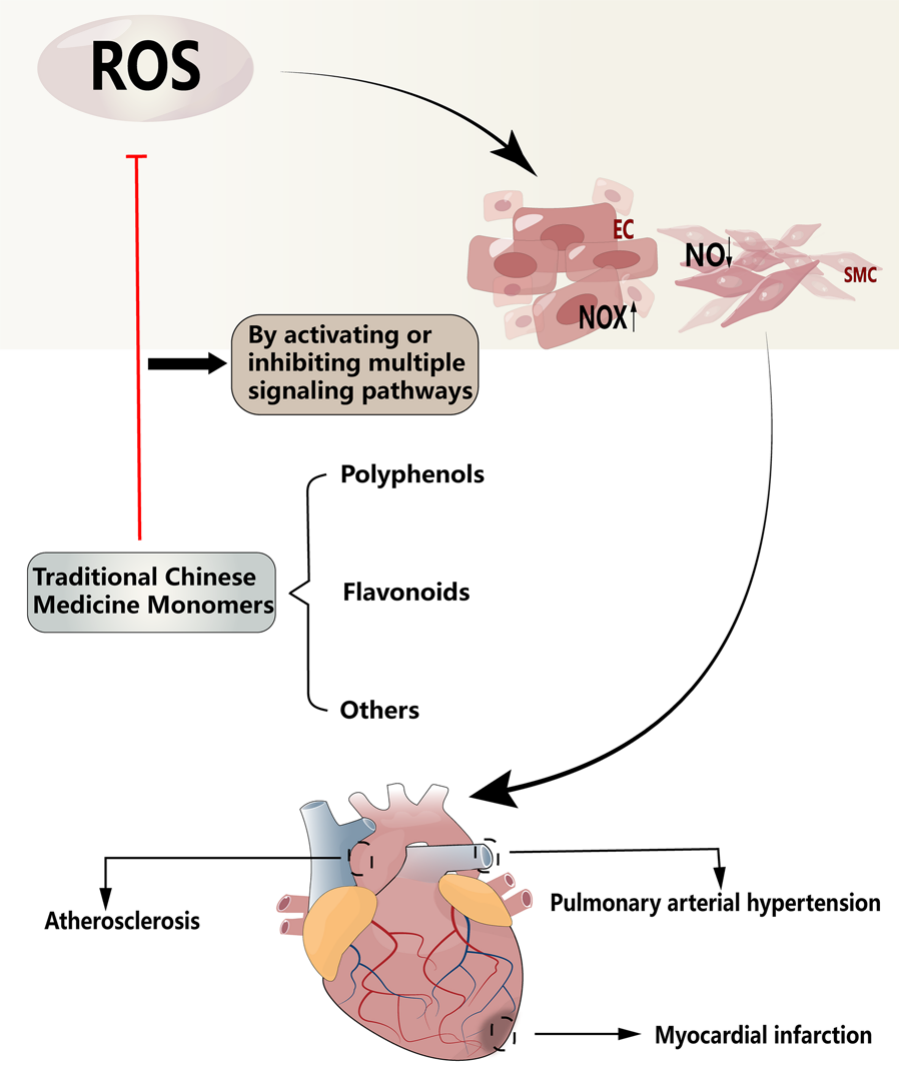

## Introduction

The term “oxidative stress” was initially introduced by Helmut Sies in 1985. The delicate equilibrium between ROS and antioxidants is crucial for the preservation of normal cellular functioning and survival [1]. Disruption of the delicate balance between them can result in detrimental consequences, leading to impaired normal cell function.

OS has been demonstrated to contribute to the pathogenesis of numerous diseases, including CVDs [2], type 2 diabetes [3], and autoimmune disorders [4]. Optimal cellular function necessitates the presence of low concentrations of ROS to facilitate cellular homeostasis and stability. However, when ROS levels surpass physiological thresholds, detrimental effects on heart cell function, as well as protein and lipid peroxidation, and DNA damage can occur [5]. The detrimental consequences of excessive ROS accumulation are notably observed in the context of cardiac health. Perturbations in ROS levels and alterations in nitric oxide (NO) signaling pathways contribute to the proliferation of pulmonary artery endothelial cells (ECs) and pulmonary vascular smooth muscle cells (PASMCs), thereby fostering the development and progression of PAH [6]. The excessive production of ROS expedites the progression of AS and facilitates the formation of atherosclerotic plaques [7]. In addition, it protects cardiac function in myocardial infarction (MI) by controlling mitochondrial bioenergetics and ROS flux [8].

Given the significant involvement of ROS signaling in the pathogenesis of CVDs such as PAH, AS, and MI, it becomes crucial to address the development of CVDs by implementing effective antioxidant strategies.

This paper provides a concise overview of the mechanisms of OS and its implications in CVDs. It discusses the sources of ROS and explores the intricate relationship between ROS signaling pathways and cardiovascular health. Moreover, the paper examines the significance of endogenous antioxidant treatments and their influence on signaling pathways. Additionally, it offers a brief summary of the signaling pathways associated with polyphenols, flavonoids, and other herbal monomers, highlighting their potential therapeutic applications in cardiovascular disease. Overall, this review aims to present a logical and scholarly analysis of the intricate interplay between OS, CVDs, and the potential use of various herbal compounds as treatments. It is expected that a better understanding of the mechanisms of action between OS and antioxidant oxygen can provide new research ideas to improve therapeutic interventions for CVDs.

## OS and ROS

Mammalian cells heavily depend on a cascade of redox reactions to generate energy, thereby facilitating the synthesis of vital cellular constituents essential for sustaining their biological functions [9]. In the initial stages, cellular redox reactions were commonly denoted as 'redox signaling' and 'redox control', encompassing their crucial role in intracellular communication and regulation. Subsequently, the concept of OS was expanded to incorporate the significant contribution of redox signaling pathways [10]. OS can be induced by various factors, both endogenous and exogenous [1]. Examples of these factors include exposure to cold temperatures, ionizing radiation, high sugar levels, cigarette smoking, and environmental pollution. ROS, which are oxygen-containing molecules, play a crucial role in OS by readily reacting with other cellular molecules [11]. ROS are generated as byproducts of oxygen metabolism and adenosine triphosphate (ATP) production during cellular respiration [12, 13]. ROS production occurs in various cellular locations, including the cytoplasm, cell membrane, endoplasmic reticulum (ER), mitochondria, and peroxisomes, as a consequence of numerous enzymatic reactions [14]. Under physiological conditions, ROS originate from various sources, highlighting the complexity of their production. Notable contributors to ROS generation encompass the mitochondrial respiratory chain, nicotinamide adenine dinucleotide phosphate (NADPH) oxidase, xanthine oxidase (XO), nitric oxide synthase (NOS), lipoxygenase, and cyclooxygenase. The majority of ROS, approximately ninety percent, are generated by the mitochondrial respiratory chain [11]. This observation underscores the prominent role of the mitochondrial electron transport chain (ETC) in ROS production. The mitochondrial ETC operates through a sequential series of electron transfer reactions, facilitating oxidative phosphorylation and the subsequent generation of cellular ATP [15]. A prominent mechanism through which ROS are generated by the ETC involves the untimely leakage of electrons from complexes I, II, and III [16]. This electron leakage leads to the reduction of molecular oxygen, resulting in the formation of superoxide (O2·^−^). Subsequently, O2·^−^ can be converted into hydrogen peroxide (H_2_O_2_).

A widely accepted notion is that OS resulting from an imbalance between excessive ROS and reactive nitrogen species (RNS) plays a significant role in the development of numerous physiological diseases, including CVDs. However, recent investigations have shed light on the paradoxical nature of ROS, highlighting their essential involvement in fundamental biological processes such as cell proliferation and differentiation [17, 18].

ROS encompass a broad spectrum of chemical species, encompassing both oxygen radicals and nonradicals. Examples of oxygen radicals include O_2_·^−^, hydroxyl radicals (·OH), and peroxyl radicals, while nonradicals encompass H_2_O_2_, hypochlorite, and ozone [5]. The mechanism underlying OS primarily revolves around the chemical reactions involving O_2_·^−^, H_2_O_2_, and ·OH. Ubiquinone, a mobile component of the mitochondrial ETC, exhibits the unique ability to function as a pro-oxidant. Within the ETC, ubiquinone participates in electron transfer reactions, including the reduction of O_2_ via single-electron transfer, resulting in the generation of O_2_·^−^ due to electron leakage [1, 19]. In addition to the mitochondrial electron transport chain, ROS can also be generated through the catalytic reaction of NADPH oxidase (NOX) [20]. NOX plays a crucial role in the production of ROS by reducing O_2_ to H_2_O_2_ through the donation of a second electron to O_2_·^−^ [1, 21]. H_2_O_2_, serving as a messenger molecule, assumes a pivotal role in redox sensing, signaling, and redox regulation, with its production primarily stemming from the breakdown of O_2_ through a swift and efficient reaction [22]. The classic Fenton reaction involves the generation of highly reactive ·OH through the interaction between specific transition metal ions, particularly Fe^2+^, and H_2_O_2_ [23]. ·OH, which are endogenously generated within the body, exhibit high electrophilicity and reactivity, enabling their interaction with a wide range of organic biomolecules [24]. This high reactivity makes ·OH capable of initiating a chain reaction of lipid peroxidation, ultimately contributing to OS [1].

## Sources of ROS

### NOXs

NADPH, as an indispensable and fundamental electron donor and cofactor, plays a crucial role in all organisms by providing the necessary reducing power for assimilation reactions and maintaining redox homeostasis [25]. The NOX family is responsible for catalyzing the reduction of O_2_ to ROS, specifically O_2_·^−^ or H_2_O_2_, which is coupled to the oxidation of NADPH. NADPH oxidases (NOXs) are a family of membrane-bound enzymes that specialize in generating ROS within the cytoplasm. This enzyme system comprises seven identified isoforms: NOX1, NOX2, NOX3, NOX4, NOX5, dual oxidase 1(Duox1), and Duox2 [26, 27]. These members are considered homologs of the phagocytic NADPH oxidase gp91phox/NOX2 [28]. Superoxide, which is generated through the activity of the NADPH oxidase system, plays a critical role in the microbicidal function of phagocytes by facilitating the transfer of electrons from cytoplasmic NADPH to molecular oxygen at the phagocytic vesicle wall [29]. The NOX (1–5) enzymes exhibit a characteristic structural organization, comprising a single cell membrane dehydrogenase domain and six transmembrane hemocyanin-coordinated domains, which span the membrane. The dehydrogenase component of NOX enzymes is composed of two lobes, each responsible for binding a specific moiety. One lobe binds the flavin adenine dinucleotide cofactor (FAD), while the other lobe binds the NADPH substrate. The structural architecture of Duox1/2 encompasses several key components. These include the N-terminal peroxidase homology domain, a distinctive transmembrane helix, and the plexiform homology domain. Additionally, Duox1/2 possesses two calcium-binding EF-hand domains, which are identical to the calcium-dependent NOX5 [30].

### Endothelial NOS

NOS serves as an additional source of cytoplasmic ROS and is predominantly located in ECs. The elevation of intracellular Ca^2+^ concentration triggers the binding of NOS heterodimers to calmodulin, which, in turn, promotes the entry of electrons into the oxygenating enzyme's structural domain [31]. The NOS system encompasses three distinct subtypes: endothelial NOS (eNOS or NOS3), neural NOS (nNOS or NOS1), and inducible NOS (iNOS or NOS2) [32]. Among these subtypes, endothelial function plays a pivotal role in maintaining vascular homeostasis, primarily through the regulation of physiological levels of NO produced by eNOS. The uncoupling of eNOS results in a reduction of NO bioavailability, leading to endothelial dysfunction, which is a characteristic feature observed in various CVDs. Endothelial dysfunction is associated with pathological conditions such as vasoconstriction, thrombosis, and inflammatory states [33]. Importantly, uncoupled eNOS has been identified as a significant source of ROS in diverse CVDs conditions. The excessive production of ROS following endothelial dysfunction further induces oxidative stress, which can be attributed to the direct interaction between free radicals and NO [34]. eNOS uncoupling can transpire through diverse mechanisms, including monomer formation, NOS phosphorylation, and inadequate levels of tetrahydrobiopterin (BH4) and L-arginine [35]. One common observation in these mechanisms is the imbalance between low levels of BH4 and its oxidized form, 7,8-dihydrobiopterin (BH2). In the presence of an NOS dimer, two BH4 molecules serve as cofactors, with their binding proportionally related to the dimeric structure. The reduced BH4 facilitates the oxidation of L-arginine, thereby promoting proper enzymatic function [35]. Furthermore, it is worth noting that both BH4 and its oxidized form, BH2, have the capability to bind to eNOS, resulting in the production of O_2_- without the concurrent generation of NO [36]. A deficiency of BH4 disrupts the ability of eNOS to maintain vascular homeostasis and facilitates the accumulation of tricarboxylic acid (TCA) cycle metabolites, namely succinate and fumarate. The increased levels of these metabolites contribute to the generation of free radicals, ultimately leading to the development of CVDs [37].

Unlike eNOS, iNOS is capable of generating higher levels of NO. However, an excessive abundance of NO can have detrimental effects on normal cardiovascular function, and healthy vascular cells typically do not express significant levels of iNOS. Nevertheless, certain pathological conditions have been associated with an increase in iNOS activity within the vasculature, as indicated by some research studies [38]. Additionally, it is noteworthy that the activation of iNOS and the subsequent production of NO may play a beneficial role in preventing ischaemia/reperfusion injury. While nNOS is primarily associated with neurons, it has also been identified in various cell types within the cardiovascular system, including vascular smooth muscle cells (VSMCs), exogenous fibroblasts, ECs, and cardiomyocytes. In these diverse cellular contexts, nNOS contributes to the homeostasis of the cardiovascular system. Correspondingly, NO produced by nNOS exerts a protective function in the pathogenesis of CVDs [39].

### ER

The ER is a highly intricate network of tubular structures present within the cytoplasm, encompassing membranes responsible for protein, steroid, and lipid synthesis and modification. It serves as a prominent organelle involved in protein synthesis [36]. Perturbations and disturbances to the ER environment can result in the accumulation of unfolded and misfolded proteins within the ER, leading to disruption of protein homeostasis, a condition known as ER stress. In response to ER stress, cells activate the unfolded protein response, a mechanism that aims to alleviate the burden of unfolded or misfolded proteins by reducing their abundance or promoting their elimination [40]. It is important to note that ROS are byproducts of protein folding, and both unfolded and misfolded proteins can contribute to an excessive production of ROS, thereby triggering oxidative stress [41].

### Mitochondria

Mitochondria, being the primary consumers of cellular oxygen, play a pivotal role in the generation of ROS, along with NOX enzymes. The production of ROS within mitochondria primarily arises from the oxidation of metabolic intermediates within the ETC, leading to the formation of superoxide. This process involves the transfer of electrons through the ETC from NADH and FADH2, resulting in the establishment of a proton gradient that drives ATP production [27]. The mitochondrial respiratory chain comprises four major complexes, namely NADH-coenzyme Q reductase (complex I), succinate coenzyme Q reductase or succinate dehydrogenase (complex II or SDH), panthenol cytochrome c reductase (complex III), and cytochrome c oxidase (complex IV or Cyt c oxidase) [42]. It is worth noting that ROS generated by complexes I, II, and III are now recognized as significant pathways involved in physiological cell signaling.

Mammalian complex I represents the most intricate enzyme within the mitochondrial ETC and serves as the principal entry point for electron entry into this respiratory pathway. Its primary function is to connect the redox reactions of NADH and ubiquinone to the process of proton pumping. Complex I facilitates the conversion of stored energy into a proton gradient across the mitochondrial membrane, which drives the synthesis of ATP. This enzymatic complex catalyzes the coupling of electron transfer from NADH to the electron carrier quinone while concurrently translocating protons across the inner mitochondrial membrane. This process can be referred to as the translocation coupling of electron transfer from NADH to quinone with the concomitant transfer of protons across the mitochondrial membrane [43, 44]. In 1979, Takeshige et al. conducted a pioneering study that demonstrated the capacity of complex I to generate ROS. This ROS production was hypothesized to result from interactions between the flavin mononucleotide cofactors and the NADH oxidation sites within complex I. Traditionally, oxidative phosphorylation-derived complexes I and III have been regarded as the primary sources of mitochondrial ROS. However, recent investigations have revealed the significant role of complex II as an additional source of ROS. Human complex II comprises four subunits (SDHA, SDHB, SDHC, and SDHD), with SDH (the substrate for complex II) also participating in the TCA cycle. In this context, complex II facilitates the oxidation of succinate to fumarate [45]. Under conditions of high succinate concentrations, complex II induces the entry of electrons into complex I through a process known as reverse electron transfer, resulting in the generation of ROS. This phenomenon occurs indirectly as a consequence of the electron flow. Conversely, at low succinate concentrations, the succinate molecule donates electrons to FAD, leading to the formation of FADH2. Subsequently, FADH2 can bind to the succinate oxidation site within complex II, directly generating ROS [46]. Complex III, also known as the cytochrome bcl complex or CoQ-Cyt c reductase, mediates the transfer of electrons derived from the reduction of QH2 through the ETC. These electrons are subsequently passed to cytochrome c. It is noteworthy that leakage of electrons from cytochrome c can occur, leading to the production of ROS [16]. Complex III exhibits a remarkable characteristic in that it is capable of releasing ROS into both the mitochondrial matrix and the intermembrane space. This property provides a more straightforward pathway for the generated ROS to enter the cytoplasm, thus contributing to their dissemination [47].

In contrast, complex IV, also referred to as Cyt c oxidase, distinguishes itself from the preceding three complexes in the mitochondrial respiratory chain by not directly generating ROS. Instead, its primary function lies in catalyzing the transfer of electrons from cytochrome c to the final electron acceptor, O_2_, leading to the production of H_2_O. This process is coupled with proton translocation, which facilitates ATP synthesis [48].

### Peroxisome

**Fig. 1:**
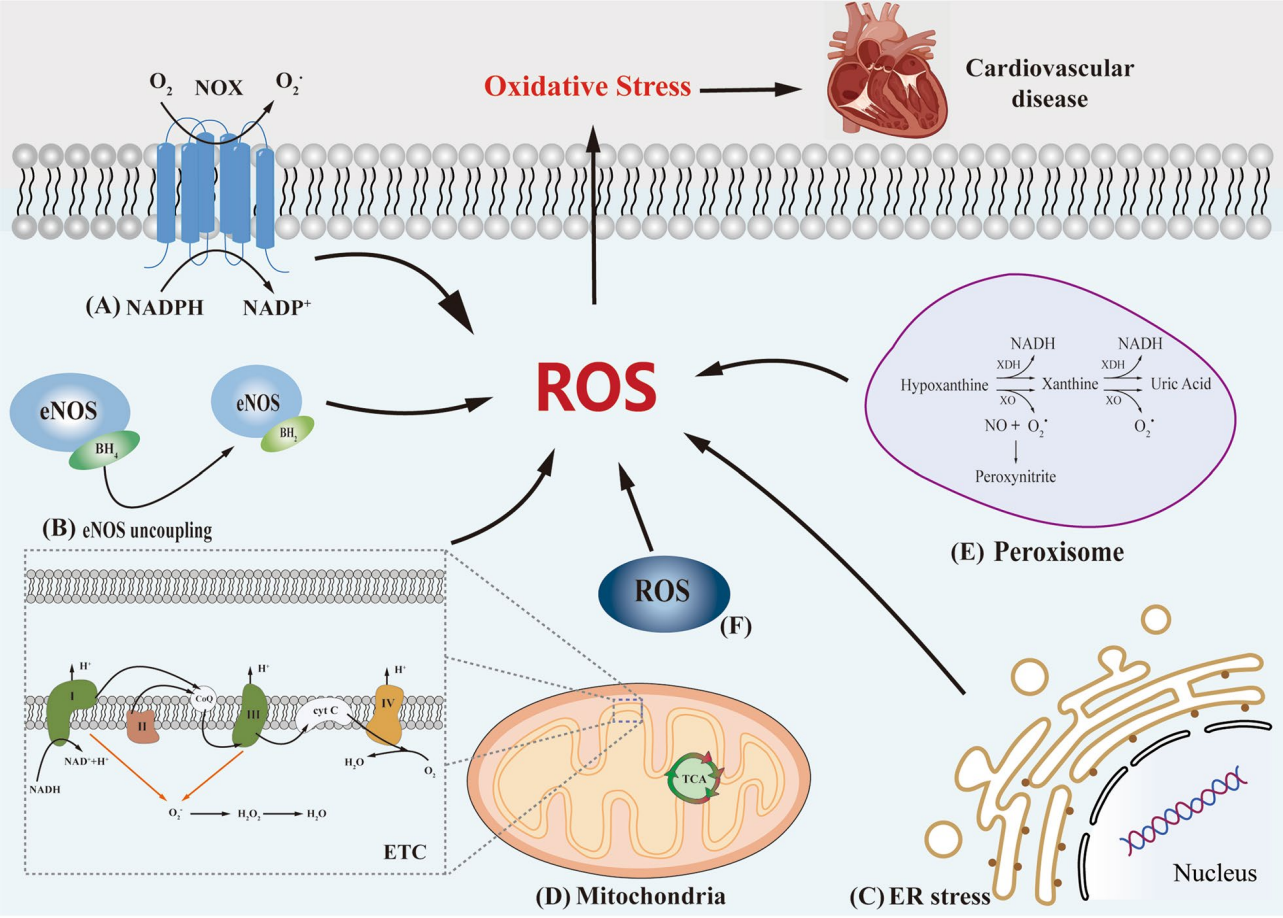
The five pathways of ROS origin are briefly described. **A** NOXs catalyze the transfer of electrons from NADPH to the plasma membrane, and then reduction of O2 generates O_2_·^−^, O_2_·^−^ as a precursor of ROS produced by cells. **B** Deficiency of BH_4_ leads to uncoupling of eNOS, which in turn causes reduced NO bioavailability. **C** After ER stress, both unfolded and misfolded proteins can lead to excessive production of ROS. **D** Complex I, II and III directly induce increased ROS expression in the mitochondrial respiratory chain causing mitochondrial dysfunction, Complex IV does not directly produce ROS in the mitochondrial respiratory chain but catalyzes electron transfer from Cyt c. **E** During the normal catalytic activity of peroxisomes, ROS is produced as a metabolic byproduct. **F** The concept of "ROS-induced ROS release" exists in ETC during ROS production, a process originally described as the production or release of ROS within an organelle that triggers enhanced ROS production or release by another compartment or organelle, and later described as extending to communication between mitochondria and plasma membrane-localized NOXs

Peroxisomes, which are single-membrane organelles crucial for the aerobic metabolism of eukaryotic organisms and lack DNA, play a significant role in the generation of ROS along with mitochondria. Notably, the peroxisomal matrix contains substantial quantities of H_2_O_2_, thus earning its nomenclature as an integral player in H_2_O_2_ metabolism. Within the peroxisome, the enzymes XO and xanthine dehydrogenase (XDH) operate synergistically to facilitate the production of superoxide radicals. This coordinated enzymatic activity relies on the interdependence of XO and XDH, thereby contributing to the generation of ROS in the peroxisomal environment. Following its involvement in purine catabolism, XDH plays a pivotal role by catalyzing the oxidation of hypoxanthine to xanthine, and subsequently, the conversion of xanthine to uric acid [49]. Furthermore, the peroxisome may also serve as a site for the generation of superoxide radicals, which could potentially arise from the short electron chain associated with the NADH/NADPH-driven peroxisomal membrane [50]. During the normal catalytic activity of various peroxisomal enzymes, ROS or RNS are produced as byproducts of metabolism [51]. These enzymes, including acyl-CoA oxidase XO and urate oxidase, generate H_2_O_2_ during the oxidation of their substrates due to the significant consumption of O_2_ by the oxidase. Although peroxisomal electron transfer does not yield ATP, excess electrons are transferred to H_2_O to generate H_2_O_2_ and NO through the action of NOS [14] (Fig. 1).

## OS signal transduction in PAH, AS and MI

### A brief review of PAH, AS and MI

Based on the latest clinical studies, pulmonary hypertension (PH) is currently categorized into five distinct classifications. Among these, pulmonary arterial hypertension (PAH) represents a subtype of PH. PAH is characterized by elevated pulmonary artery pressure attributed to augmented pulmonary vascular resistance. This condition manifests through several key features, including dysfunction of the pulmonary artery endothelial cells (PAECs), uncontrolled proliferation of PASMCs, and extensive vascular remodeling. In severe cases, these pathological changes can lead to right heart failure and ultimately result in fatality. The delineation of these characteristics contributes to a comprehensive understanding of PAH, enabling improved clinical management and therapeutic interventions. OS plays a crucial role in the pathophysiology of PAH. The production of ROS and RNS within the pulmonary circulation has been shown to modulate the disease process in PAH. Early speculations suggested that ROS could act as mediators of hypoxic constriction, potentially exerting vasoconstrictive effects on the pulmonary circulation. Conversely, the release of RNS from the vascular endothelium was hypothesized to inhibit and buffer hypoxic pulmonary vasoconstriction [52].

AS is a complex, multifactorial chronic inflammatory vascular disease characterized by the gradual accumulation of fatty and/or fibrous materials within the innermost layer of the artery, known as the intima [53]. The pathogenesis of AS involves a sequence of events, beginning with endothelial injury followed by the deposition of lipid material within the atherosclerotic plaque. Over time, this process leads to the proliferation of fibrous tissue and the accumulation of calcium material, ultimately culminating in the occurrence of acute cardiovascular events [54]. In addition to the traditional belief linking the oxidation and elevated levels of low-density lipoprotein (LDL) cholesterol to the pathogenesis of AS, emerging evidence suggests that the retention and accumulation of triglyceride-rich lipoprotein residues within the arterial intima contribute to abnormal lipid deposition and promote the development of AS [55]. OS serves as a pivotal factor in the formation of atherosclerotic plaques, exerting multifaceted effects on the pathophysiology of AS. OS triggers ECs proliferation, VSMCs proliferation, and vasoconstriction, ultimately leading to endothelial dysfunction and the initiation and progression of AS [56].

MI is defined pathologically as the death of cardiomyocytes resulting from chronic myocardial ischemia, characterized by a reduced oxygen supply to the myocardium [57]. The occurrence of MI is often precipitated by the rupture or erosion of fragile atherosclerotic plaques, which, when combined with coronary thrombosis, can lead to detrimental consequences. In severe cases, this can cause damage to the coronary microcirculation and result in complete coronary occlusion [58]. MI can give rise to various complications, including heart failure, heart remodeling, and myocardial ischemia–reperfusion(I/R) injury. Notably, OS plays a pivotal role in the pathophysiology of MI. Both myocardial ischemia and reperfusion, with or without subsequent reoxygenation, can induce the generation of ROS. In the context of ischemic myocardium, particularly following reperfusion, ROS can directly impair cell membranes, leading to cellular death and significant damage to the heart [59]. Firstly, the cardiomyocyte necessitates a rapid and continuous resynthesis of ATP to satisfy the high-energy phosphate demands required for maintaining cardiac contractile function and ion transport. The majority—over 95%—of ATP production occurs within the mitochondria through oxidative substrate utilization. Secondly, following MI, myocardial ischemia is intensified with prolonged ischemic durations. However, the introduction of early reperfusion exacerbates myocardial damage. Notably, myocardial ischemia prompts alterations in mitochondrial structure and function, rendering them more susceptible to excessive production of ROS during ischemic reperfusion events [59].

### Endothelial dysfunction induces OS aggravate PAH, AS and MI

In patients with PAH, the endothelial dysfunction signaling exhibits several distinctive features, including lung inflammation, OS, excessive proliferation, and the accumulation of inflammatory cells and fibroblasts [60]. Notably, the impairment of pulmonary vascular endothelial function contributes to OS, primarily attributed to diminished bioavailability of NO and increased degradation of NO, while eNOS uncoupling further promotes ROS production [61]. NO exhibits potent vasodilatory effects within the pulmonary circulation and plays a crucial role as an antioxidant in cellular signaling. The arginine-NOS/NO pathway assumes a vital role in regulating vascular tone and pulmonary artery remodeling in the context of PAH [6]. Yu WC et al. demonstrated that the carbonic anhydrase 1-kininogen and selenoprotein W/143–3 signaling pathways mitigated the suppression of eNOS activity and facilitated NO production in a rat model of monocrotaline-induced PAH [62]. Furthermore, NO production not only attenuated oxidative stress but also counteracted vasoconstriction and vascular cell proliferation. These effects culminated in improved pulmonary hemodynamics, reduced pulmonary vascular resistance, and ultimately ameliorated PAH [63, 64].

AS shares similarities with PAH in terms of endothelial dysfunction, which leads to a reduction in the bioavailability of NO and an increase in NO degradation, thereby causing OS. ROS play a significant role in all stages of inflammation in AS, as the early phase of AS is characterized by intricate interplay between OS and inflammation. In response to oxidative damage, inflammatory processes may attempt to repair the damage, which can induce further OS and ROS production. The primary redox-sensitive transcription factor involved in AS is nuclear factor-κB (NF-κB), which not only promotes ROS production but also induces the synthesis of proinflammatory cytokines such as tumor necrosis factor-alpha (TNF-α) and activates NF-κB itself. Consequently, ECs stimulate the synthesis of inflammatory factors and upregulate the expression of adhesion molecules, ultimately leading to increased infiltration of immune cells into the diseased vessel site, thereby exacerbating the vascular inflammatory response [65, 66]. eNOS is predominantly expressed in ECs and plays a crucial role in producing NO. NO production by eNOS exerts various beneficial effects, including inhibition of LDL oxidation, prevention of leukocyte adhesion and migration, suppression of VSMC proliferation, and inhibition of platelet aggregation. Furthermore, enhanced eNOS activity contributes to improving endothelial dysfunction and mitigating the development of AS [67]. The imbalanced activation of NOS isoforms expressed by ECs, particularly eNOS, contributes to endothelial dysfunction, which further promotes the activation of the inducible subtype of the enzyme, iNOS. This augmented iNOS activity enhances inflammatory processes within the vascular walls, thereby contributing to the progression of AS [34]. In contrast, the upregulation of nNOS may confer vasodilatory effects and exert antiatherosclerotic properties. This upregulation of nNOS can partially compensate for the loss of H_2_O_2_ derived from NOX4 and help maintain endothelial homeostasis [68].

Impaired ECs following MI compromise the structural integrity and microcirculatory function of the coronary microvascular system. The generation of ROS by NOX enzymes contributes to the disruption of the coronary microvascular system's structure and exacerbates coronary microvascular injury [69]. Moreover, ROS-induced oxidation of endothelial-derived NO leads to the formation of peroxynitrite, triggering vasoconstriction and diminishing the bioavailability of NO, which otherwise contributes to vasodilation [69]. CO exposure induces the overexpression of NOS and subsequent excessive production of NO, along with increased ROS generation. This dysregulated production of NO and ROS can have detrimental effects on the heart [70]. Among the NOS isoforms, iNOS predominantly produces substantial amounts of NO in the heart, while nNOS and eNOS contribute to lower levels of NO production in the cardiac tissue [70]. The iNOS/NO signaling pathways have been implicated in the pathogenesis of myocardial ischemia–reperfusion injury (MIRI) and are known to exacerbate cardiac injury and myocardial infarction by promoting OS [70]. However, emerging evidence suggests a protective role of NO overexpression mediated by iNOS in the context of MIRI, potentially contributing to ischemia-induced late preconditioning against vertigo and infarction. The myocardial protection conferred by iNOS may involve the hypoxia-inducible factor-1 alpha (HIF-1α) signaling pathway, leading to the opening of mitochondrial ATP-sensitive K(+) channels and elevated levels of TNF-α and cyclooxygenase-2 (COX-2)-dependent prostaglandins [71, 72]. Similar to iNOS, studies investigating the role of nNOS and eNOS in the context of MIRI have provided evidence for their protective effects on cardiac function, including the prevention of cardiac remodeling following MI, as well as the potential inhibition of superoxide production [73, 74]. Furthermore, nicorandil, a pharmacological agent, has shown promise in improving coronary microvascular function in patients with acute MI by modulating the signaling pathway of eNOS [75].

### NOXs induce OS to aggravate PAH, AS and MI

**Fig. 2:**
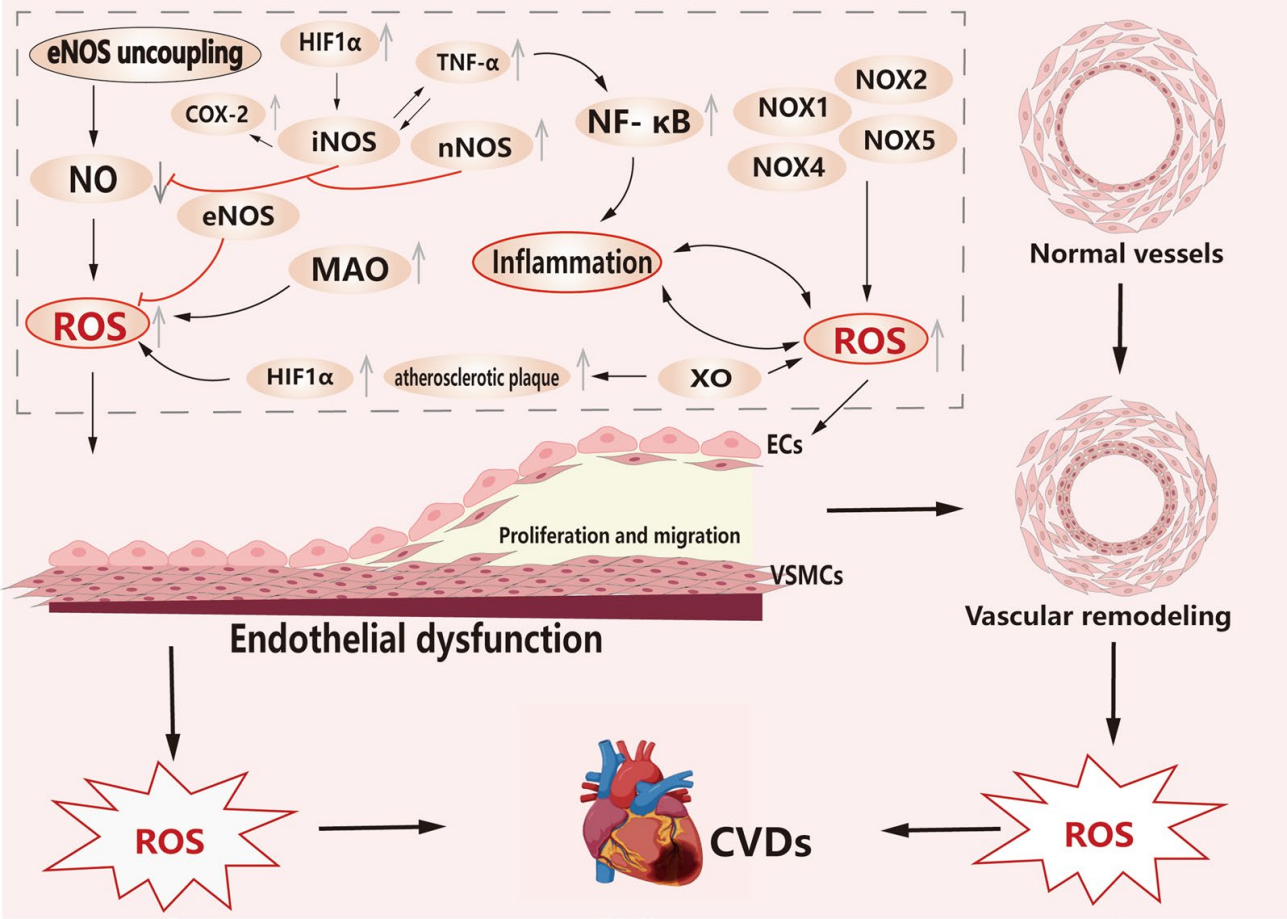
Overproduction of ROS leads to endothelial dysfunction, which can be seen in the proliferation and migration of PAECs and PASMCs. Endothelial dysfunction and ROS overproduction are also potential factors that exacerbate vascular remodeling. The uncoupling of eNOS, the activation of NOXs, and the inflammatory response lead to an increase in ROS expression, which in turn promotes the development of CVDs. In ROS-induced disease processes, disease progression also results in more ROS production, which further exacerbates the disease. In addition, XO induced the production of ROS, which increased the accumulation of atherosclerotic plaques. MAO plays an important role in the MIRI process and is an important source of mitochondrial ROS in myocardial metabolic pathology. MAO promotes the generation of ROS. (black arrows represent promotion, red arrows represent inhibition, and gray arrows represent elevated or reduced expression)

There is substantial evidence supporting the significant impact of NOXs on the pathogenesis of PAH when utilized as a source of ROS. Specifically, when NOXs serves as the primary ROS source, it can modulate cell signaling, leading to the induction of PASMCs proliferation in various PAH models. Moreover, studies have shown that NOX1 expression deficiency in mice with hyperoxia-induced acute lung injury phenotype can attenuate acute lung injury [76]. In human pulmonary ECs, the primary ROS sources comprise NOX1, NOX2, and NOX5, which are predominantly expressed as superoxide generators. Additionally, NOX4 is predominantly overexpressed as a hydrogen peroxide generator [6]. Notably, in an experimental study involving serotonin-induced NOX1 production and ROS, the NOX1 pathway was found to contribute to the proliferation and vascular remodeling of human PASMCs in the context of PAH [77]. NOX1 plays a crucial role in promoting vasoconstriction and elevated blood pressure through the facilitation of calcium signaling in VSMCs in response to angiotensin II [78]. In human PAECs, the activation of the NOX1-protein kinase A-cyclic-AMP response binding protein/redox factor-1 signaling pathway, under conditions of in vitro hypoxia exposure, leads to the production of ROS. This, in turn, stimulates ECs proliferation, migration, and vascular remodeling in the context of PAH [79]. Furthermore, stimulation of human ECs with VEGF results in rapid cellular oxidation that persists for a minimum of 60 min. Inhibition of NOX2 and NOX4 expression significantly hampers VEGF-induced tyrosine phosphorylation of the type 2 VEGF receptor, along with ECs migration and proliferation [80]. Knockdown of NOX4 in pulmonary microvascular ECs has been shown to effectively reduce ROS production in response to lipopolysaccharide (LPS) stimulation. This provides compelling evidence for the role of NOX4 in inducing dysfunction and injury in the lung ECs barrier. Moreover, the study demonstrated that NOX4-mediated redox-sensitive activation of the calmodulin-dependent protein kinase II (CaMKII)/ERK1/2/myosin light chain kinase pathway plays a significant role in this process, as observed in a preclinical model of sepsis induced by cecal ligation puncture [81]. Furthermore, in transgenic mice with NOX1 deficiency, pulmonary vascular remodeling was observed. Additionally, the inactivation of NOX1/NADPH oxidase resulted in an imbalance in PASMCs turnover and subsequent hypertrophy of the medial pulmonary artery, underscoring the involvement of NOX1 in regulating pulmonary vascular remodeling [82]. Moreover, angiopoietin II (Ang II) has been found to induce PASMCs activation through the NOX2-ROS pathway. This pathway demonstrates the critical role of NOX2 in mediating Ang II-induced effects on PASMCs, further emphasizing the importance of NOX isoforms in PAH pathogenesis [83]. In a comprehensive study investigating the impact of the estrogen metabolite 16α-hydroxyestrone (16αOHE1) on PASMCs, notable findings revealed its role in promoting increased NOX expression, leading to ROS production and subsequent proliferative responses in PASMCs, thereby facilitating vascular remodeling. Specifically, the study demonstrated that 16αOHE1 significantly upregulated the expression of NOX1 and NOX4, with a more pronounced effect on NOX1 induction. Additionally, 16αOHE1 stimulation resulted in protein tyrosine phosphatase oxidation, concurrent with a decrease in nuclear factor erythroid-2-related factor 2(Nrf2) activity and antioxidant gene expression, ultimately culminating in enhanced proliferation of HPASMCs. Notably, the study also employed female mice, revealing that the upregulation of the NOX1/ROS/Nrf2 signaling pathway might play a crucial role in vascular damage associated with PAH, particularly in female subjects [84]. Furthermore, the study's investigation into the influence of 5-HT on PASMCs uncovered its role in inducing migration and proliferation via the activation of NOX4-ROS-TRPM2 signaling pathway [85]. Activation of ROS and HIF1α in a hypoxic model of PH has been shown to promote proliferation of PASMCs, leading to pulmonary vascular remodeling and the development of PH [86]. Furthermore, NADH dehydrogenase (ubiquinone) 1α subcomplex 4 like 2 (NDUFA4L2) has been identified as a proliferative factor, and knockdown of NDUFA4L2 has been demonstrated to reduce the proliferation of human PASMCs under hypoxic conditions, along with mitigating excessive ROS production during hypoxia [87]. In summary, the dysregulation of endothelial-derived contractile factors, such as ET-1, and decreased levels of diastolic factors, such as NO, can exacerbate OS-mediated endothelial dysfunction. Inhibition of ROS production offers a potential avenue to mitigate endothelial dysfunction, PASMCs migration and proliferation, as well as vascular remodeling associated with PAH [88] (Fig. 2).

In the context of AS, the primary source of ROS within the blood vessel wall is attributed to NOXs. Specifically, ECs express NOX2, NOX4, and NOX5, while VSMCs express NOX1, NOX4, and NOX5 [89]. Extensive research has implicated NOX1 in the development of AS lesions, and TLR2 signaling, through the activation of NOX1, promotes a proinflammatory phenotype in mouse aortic smooth muscle cells, stimulating mouse aortic smooth muscle cells migration and vascular remodeling [90]. Furthermore, ET-1 overexpression has been shown to exacerbate perivascular OS and inflammation in diabetic AS, mediated by NOX1. Specifically, ET-1 contributes to the activation of NOX1, leading to increased OS and inflammation within the perivascular region [91]. In a separate study, dabrin, a therapeutic agent, exhibited inhibitory effects on smooth muscle cell migration, proliferation, and proinflammatory signaling. This inhibition was achieved through the suppression of NOX1 activity, thereby limiting the progression of AS [92]. The upregulation of NOX2 and its increased expression within atherosclerotic plaques have been associated with OS. Pharmacological inhibition of NOX2 has shown promise in delaying the development of AS and reducing thrombosis in platelet-associated thrombosis [93]. Additionally, a study focusing on the lysophosphatidylcholine-driven production of NOX5-dependent ROS demonstrated that NOX5 induced OS and endothelial cell dysfunction, highlighting the contributory role of NOX5 in AS [94]. However, it is important to note that the emergence of ROS in AS does not invariably promote oxidative stress. NOX4, for instance, functions as an endogenous anti-atherosclerotic enzyme, providing protection against AS by inhibiting inflammation and vascular remodeling [95, 96].

The production of ROS at complexes I and III, which are upstream of Cyt c oxidase, primarily occurs due to reverse electron transfer resulting from nonspecific electron leakage and succinate accumulation during ischemia. Among these processes, reverse electron transport at complex I represents the main source of ROS generation [97]. In the context of MI and reperfusion injury, NOX isoforms display both beneficial and detrimental effects on myocardial tissue. NOX1, NOX2, and NOX4 have been implicated in contributing to myocardial injury, while NOX5, which is deficient in rodents, has also demonstrated a role in MI and has been linked to increased ROS expression in human studies [98–100]. Interestingly, NOX4, specifically, shows upregulation in the heart following MI. Elevated NOX4 levels in cardiomyocytes have been observed to modulate intramyocardial inflammation and increase the presence of macrophages. Additionally, NOX4-driven macrophage polarization has been shown to prevent cell death and adverse remodeling in the aftermath of MI [101].

### XO induces OS to aggravate AS

XO is a crucial enzyme involved in uric acid metabolism. Accumulation of XO in atherosclerotic plaques has been associated with the generation of ROS. XO is believed to contribute to the oxidation pathway that promotes the progression of atherosclerotic plaques [102, 103]. Mitochondrial OS has emerged as a significant factor in the development and advancement of AS in humans. Nevertheless, studies have demonstrated that modulation of mitochondrial function holds potential as a therapeutic intervention for AS. For instance, upregulation of peroxisome proliferator-activated receptor-gamma coactivator-1alpha (PGC-1α) has been shown to prevent the development of AS. Moreover, macrophage-specific Dicer expression has been found to inhibit AS by promoting mitochondrial oxidative metabolism [104–106].

### MAO induces OS aggravate MI

**Fig. 3:**
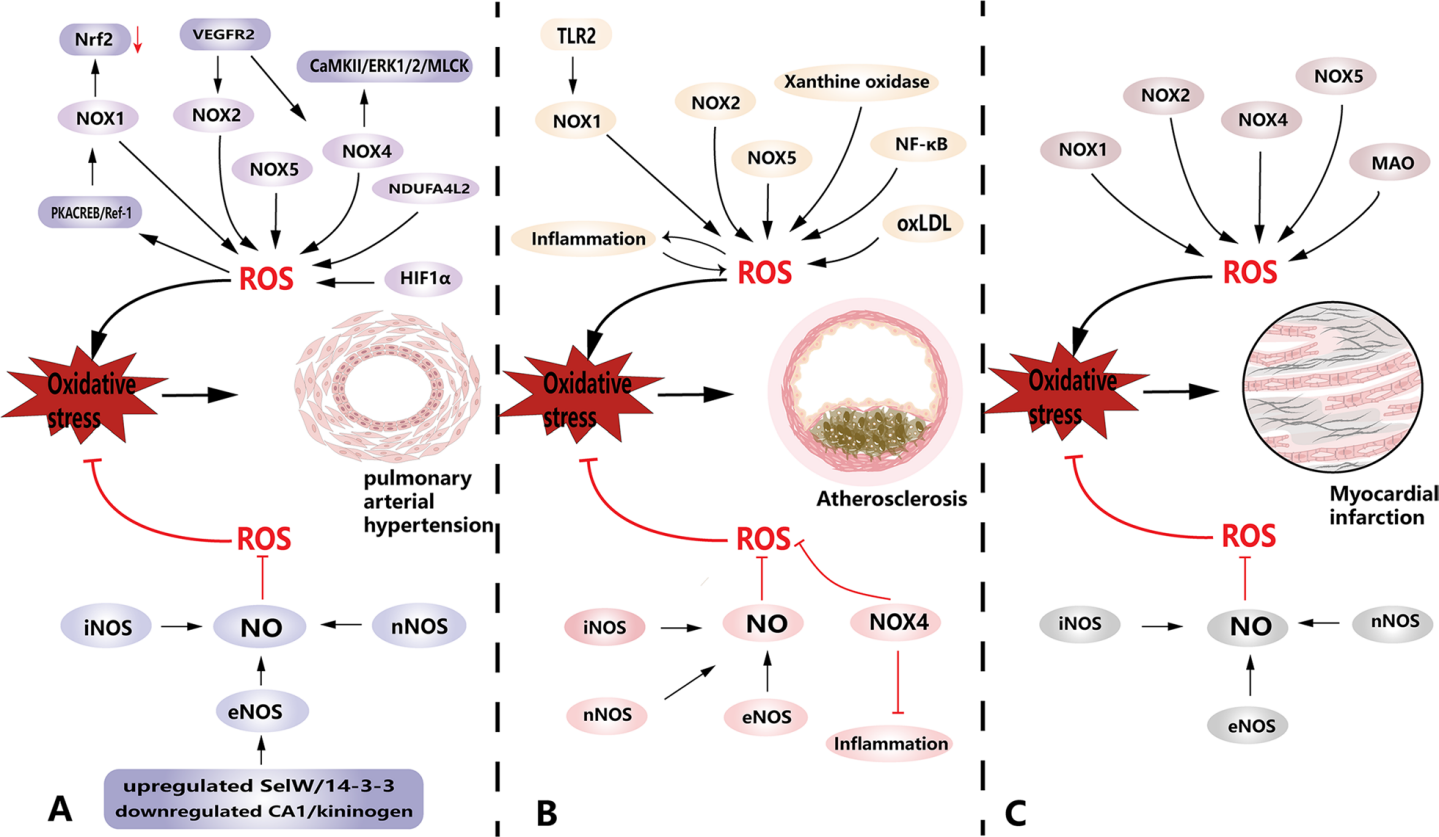
Elevated ROS can exacerbate the development of PAH, AS and MI, and inhibition of ROS expression can slow down the development of PAH, AS and MI. **A** OS promoted the development of PAH, as seen by the proliferation of PAECs and PASMCs promoting vascular remodeling compared to normal vascular architecture. NOXs and their associated signaling pathways contribute to the development of PAH, such as the NOX1/ROS/Nfr2x signaling pathway that activates and promotes OS to exacerbate disease development. In addition, HIF1α and NDUFA4L2 under hypoxic conditions promote cell proliferation exacerbating disease progression. **B** There is lipid and/or fiber accumulation in the AS vessels compared to normal vessels (visible as a gray-brown fraction), a process facilitated by OS. In addition, AS as a chronic inflammatory disease, there is crosstalk between OS-related and inflammatory signaling pathways during the development, for example, NOXs exacerbate the development of AS and perivascular inflammatory response, and the inflammatory response further drives the overproduction of ROS in an interactive process. In this process, the NF-κB signaling pathway deserves to be focused on. In addition, XO also serves as an important source of ROS to drive the development of AS. (C) Cardiomyocyte death and fibrous scarring were observed in MI compared to normal cardiomyocytes. NOXs are involved in promoting the process of myocardial injury. Large amounts of ROS are also produced after MI, further exacerbating cardiomyocyte death. Notably, NOX4 may reduce the protective effect of inflammation on cardiomyocytes. In addition, MAO induced increased ROS expression and exacerbated MI. Finally, increasing the bioavailability of NO can reduce the expression of ROS and slow down the development of PAH, AS and MI

During myocardial ischemia, a condition characterized by insufficient blood flow to the heart, certain changes occur within mitochondria. Mitochondria release Cyt c and experience a loss of cardiolipin, a phospholipid crucial for mitochondrial function. This process is exacerbated by the production of ROS during ischaemia reperfusion, leading to oxidative damage of cardiolipin. Consequently, this oxidative stress may result in cardiolipin deficiency [59]. In the context of cardiometabolic pathology, two isoforms of monoamine oxidase (MAO), namely MAO-A and MAO-B, have gained attention as significant sources of mitochondrial ROS. MAO is a protein located in the outer mitochondrial membrane responsible for catalyzing the oxidative deamination of biogenic amines, which ultimately leads to ROS production. Interestingly, the absence of MAO has been associated with reduced occurrence of irreversible myocardial ischemia and diminished myocardial injury during I/R [107, 108] (Fig. 3).

## Antioxidant treatment

Antioxidants play a crucial role in mitigating the development of various diseases, including CVDs, by counteracting the detrimental effects of ROS and preserving normal cellular physiological function. They can be classified into endogenous and exogenous antioxidants based on their source. The endogenous antioxidant systems encompass a range of enzymes, including glutathione peroxidase (GPX), superoxide dismutase (SOD), and catalase (CAT). These enzymes work in concert with nonenzymatic antioxidants such as uric acid, melatonin, glutathione (GSH), and polyamines to combat ROS and maintain cellular integrity [109]. On the other hand, exogenous antioxidants are abundantly found in natural plants and primarily consist of polyphenols and natural flavonoids. Supplementation with exogenous antioxidants exerts potent antioxidant effects by engaging various signaling pathways. These pathways include augmenting the antioxidant capacity of endogenous antioxidant systems, thereby reducing OS; inhibiting ROS production, consequently restraining OS; and activating antioxidant signaling pathways that counteract OS.

### Endogenous antioxidants

GPX is a selenium-dependent vertebrate protein that plays a crucial role in inhibiting the generation of free radicals from hydroperoxides. Besides its antioxidant function, GPX is also involved in various regulatory processes and synthetic functions [110]. The presence of GPX has been shown to have beneficial effects on anti-inflammation, prevention of vascular endothelial dysfunction, and inhibition of AS. Studies utilizing mice lacking GPX in PAECs have demonstrated that the absence of GPX leads to reduced phosphorylation of protein kinase B (Akt), heightened activity of NF-κB, and significantly increased expression of vascular cell adhesion molecules. Notably, the deficiency of GPX impacts the Akt pathway, thereby influencing eNOS-derived NO signaling [111]. Deficiency of glutathione peroxidase-1 (GPX-1) has been associated with impaired eNOS function and uncoupling, leading to endothelial dysfunction. This deficiency results in reduced bioavailability of NO, which plays a critical role in maintaining vascular homeostasis. Additionally, GPX-1 deficiency contributes to vascular remodeling, heightened OS, and facilitates leukocyte adhesion and infiltration into cardiovascular tissues, particularly in the context of animal aging [112].

Furthermore, GPx3 deficiency in patients has been linked to increased OS, endothelial dysfunction, coronary thrombosis, and diminished left ventricular function [113, 114]. Doxorubicin (DOX), a widely used anticancer drug, exerts its deleterious effects on cellular homeostasis by downregulating the expression of GPx4 and inducing excessive lipid peroxidation within mitochondria. This process is facilitated by the formation of the DOX-Fe^2+^ complex, ultimately leading to mitochondria-dependent iron death. Iron death represents an oxidative form of regulated necrotic cell death characterized by the disruption of GPx4's antioxidant capacity and the generation of ROS. In the context of cardiovascular pathologies, the expression of GPx4 plays a crucial role in mitigating myocardial damage and inhibiting iron death, thereby delaying the progression of MI and AS [115, 116]. Studies have demonstrated that the overexpression of GPx4 can effectively reduce lipid peroxidation and mitigate endothelial dysfunction, thereby suppressing the development of AS [117]. Moreover, the identification of a novel signaling pathway, Egr-1/miR-15a-5p/GPX4/iron death, has shed light on the molecular mechanisms involved in the progression of MI. Activation of this pathway leads to an increase in GPX4 protein expression, which in turn inhibits concomitant iron death and subsequent myocardial injury, consequently delaying the development of MI [118].

SOD plays a crucial role in the defense against oxygen free radicals by catalyzing the conversion of superoxide to oxygen and hydrogen peroxide. This enzyme is widely expressed in aerobic organisms, making it a key component of the primary line of defense against oxidative stress [119]. SOD exists in three distinct isoforms: cytoplasmic SOD1, mitochondrial SOD2, and extracellular SOD3. SOD2 and SOD3 are predominantly found within eukaryotic cells, with SOD3 being the primary isoform present in the cardiovascular system. Manganese superoxide dismutase, also known as SOD2, is an antioxidant enzyme encoded by nuclear genes. Disruptions or alterations in SOD2 activity have been associated with mitochondrial structural abnormalities observed in conditions such as heart failure. A deficiency in SOD2 levels leads to an accumulation of ROS and subsequent overproduction of 4-Hydroxynonenal within the mitochondria. Notably, the signaling interplay involving SOD2-mediated 4-Hydroxynonenal connections has been implicated in the origin of mitochondrial ROS production and the development of cardiomyopathy [120]. Mitochondrial dysfunction in ECs and VSMCs contributes to the progression of vascular diseases, including AS and vascular calcification. One key player in combating mitochondrial ROS in ECs and VSMCs is manganese SOD2. SOD2 possesses a selective affinity for mitochondria and plays a crucial role in mitigating ROS-induced damage. Studies have demonstrated that SOD2 inhibits signaling pathways such as Janus kinase/signal transducer and activator of transcription (JAK/STAT) and phosphoinositide 3-kinase/protein kinase B (PI3K/Akt), thereby impeding vascular senescence and delaying the progression of vascular disease [121]. On the other hand, extracellular SOD3 is a copper (Cu)-containing enzyme that exhibits notable cardioprotective effects, particularly in patients with ischemia and reperfusion injury. SOD3 has been implicated in the modulation of left ventricular structure remodeling and the subsequent development of heart failure in patients with cardiovascular disease [122, 123]. Caveolin-1, an essential structural protein in caveolae, has been shown to positively modulate the activity of extracellular SOD3 by promoting the stabilization of ATP7A protein expression. ATP7A serves as a copper transporter protein responsible for the delivery of copper to SOD3, facilitating its enzymatic function. This mechanism contributes to the prevention of endothelial dysfunction mediated by vascular OS [124]. Moreover, emerging evidence suggests that SOD3 may exert protective effects in the context of PAH and cardiac function regulation. Under stressful conditions, SOD3 has been implicated in safeguarding the lungs against PAH development. Furthermore, it appears to play a role in maintaining normal heart morphology, reducing cardiac myocyte hypertrophy, cardiac remodeling, and mitigating inflammation [125–127]. CAT plays a crucial role in protecting the body against OS. Overexpression of CAT in the heart has been shown to effectively reduce intracellular ROS levels, improve mitochondrial structural abnormalities, and contribute to the maintenance of normal cardiac function [128]. Elevated levels of SOD and CAT in the heart have demonstrated varying degrees of efficacy in enhancing plasma antioxidant enzyme activity and reducing lipid levels, thereby mitigating AS associated with OS [129]. Notably, under hypoxic conditions, the protein silencing information regulatory factor 6(SIRT6) has been implicated in promoting angiogenesis within plaques by regulating the activity of HIF-1α. However, it has also been observed that SIRT6 can suppress CAT activity by binding to the CAT promoter, leading to an overexpression of ROS. This dysregulation of CAT and subsequent ROS accumulation contribute to vascular injury, which in turn plays a pivotal role in the development of carotid plaque formation and bleeding, thereby contributing to the progression of AS [130]. Elevation of CAT activity has been shown to modulate OS and exhibit protective effects on the heart following acute MI induced by isoproterenol (ISO). These protective effects include a reduction in infarct size and enhancement of angiogenesis [131, 132]. In neonatal porcine PASMCs, exposure to hypoxic conditions leads to a decline in CAT activity and an increase in lipid peroxidation. This dysregulation occurs through the inhibition of the adenosine 5’-monophosphate-activated protein kinase (AMPK)-Forkhead box O (FoxO) pathway, which contributes to the production of ROS, the development of PAH, and pulmonary vascular remodeling [133].

Nonenzymatic antioxidants serve as a secondary line of defense against ROS by swiftly neutralizing free radicals and oxidants. Among these antioxidants, GSH plays a crucial role in modulating the development and progression of cardiovascular disease by influencing the intracellular redox pattern. GSH exhibits a dual role as an intracellular antioxidant. While GSH can induce oxidative stress, the magnitude of its induction effect is considerably lower compared to its potent antioxidant properties. As an antioxidant, GSH efficiently scavenges ROS and nitrogen species, either directly or indirectly, by acting as a cofactor to support the activity of various enzymes [134]. Furthermore, reduced levels of GSH trigger an imbalance in ROS levels, uncoupling of eNOS, and subsequent impairment of normal endothelial function, ultimately leading to endothelial dysfunction [135].

### Herbal monomers with powerful antioxidant capacity: polyphenols

Polyphenols, a diverse group of natural compounds abundantly present in plants, exhibit a range of biological activities that confer protection to heart function. These activities include antioxidant properties, anti-inflammatory effects, and improvements in endothelial dysfunction. Structurally, polyphenols commonly feature one or more aromatic rings interconnected by hydroxyl groups. They can be broadly classified into two main families: flavonoids and non-flavonoids [136]. Polyphenols are widely distributed in various dietary sources such as red wine, tea, fruits, and citrus fruits. Additionally, they can be derived from herbs, stems, and flowers [136, 137].

Polyphenols possess potent antioxidant capacity attributed to their ability to donate hydrogen and electrons, thereby stabilizing cellular oxidation and mitigating oxidative damage by terminating the reaction between ROS/RNS. Moreover, polyphenols play a crucial role in the defense systems of plants, aiding in reducing ultraviolet radiation and combating plant pathogens [137]. In the context of CVDs, the intricate interplay between OS and inflammatory responses cannot be overlooked. Polyphenols exert their antioxidant effects by engaging diverse signaling pathways and inhibiting the expression of NF-κB and its downstream signaling cascades. Notably, resveratrol, for instance, has been shown to confer cardioprotective effects by activating AMPK-related, Nrf2-related, and PI3K/Akt-related signaling pathways to counteract OS, while concurrently inhibiting the inflammatory response [138–140].

### Resveratrol

Resveratrol (RSV) possesses notable antioxidant and anti-inflammatory properties, which contribute to its ability to upregulate eNOS function. This upregulation of eNOS activity leads to a reduction in ROS levels, thereby alleviating OS and inhibiting lipid peroxidation. Moreover, RSV promotes NO synthesis and inhibits endothelial dysfunction, resulting in beneficial effects on CVDs such as PAH, AS, and MI [141]. The cardiovascular protective effects of RSV are mediated through multiple molecular targets [142]. For instance, RSV exhibits anti-atherosclerotic properties by reducing plasma triglyceride and LDL-cholesterol levels, inhibiting LDL oxidation, and increasing high-density lipoprotein-cholesterol [143]. Notably, RSV activates SIRT1, a class III histone deacetylase, which is considered one of the most potent naturally occurring compounds with SIRT1 activating properties. The activation of SIRT1 leads to downstream effects on the FoxO transcription factor, a crucial antioxidant factor that helps maintain endothelial morphology. The Akt pathway, regulated by SIRT1, plays a pivotal role in modulating FoxO activity. Activation of SIRT1 induces the expression of FoxO3a, which, in turn, promotes eNOS expression, thereby inhibiting intracellular ROS production and mitigating OS damage [144, 145]. The SIRT1/FoxO axis plays a crucial role in mediating the induction of eNOS by resveratrol, leading to enhanced NO production and increased eNOS expression and activity [146]. In the context of I/R injury, SIRT1 has been shown to reduce ROS production during myocardial I/R and inhibit the activation of the NLRP3 inflammatory vesicle by suppressing cardiac pyruvate dehydrogenase activation [147]. Moreover, resveratrol has been demonstrated to significantly enhance the antioxidant capacity of rat cardiomyocytes injured by hypoxia/reoxygenation, restoring mitochondrial quality control in MIRI through the SIRT1/SIRT3-mitofusin2(Mfn2)-phosphorylated Parkin-PGC-1α pathway. Additionally, its protective effect on mitochondria may be attributed to the activation of the SIRT1/SIRT3-FoxO signaling pathway [148]. Resveratrol has also been found to confer protective effects on right ventricular dysfunction and pathological remodeling in mice with monocrotaline-induced PAH, contributing to improvements in pulmonary vascular architecture. However, the extent of improvement may be limited. These beneficial effects could also be associated with the activation of specific sirtuin pathways, particularly SIRT1 and SIRT3 [149]. SIRT1 serves as a target against endothelial dysfunction and can inhibit perivascular lipid incorporation and accumulation. This process is regulated by increased phosphorylation of AMPK, which promotes SIRT expression and subsequently inhibits the expression of key regulators of adipogenesis, such as peroxisome proliferator-activated receptor γ (PPARγ), essential for adipogenesis [150, 151].

AMPK plays a crucial role in governing both catabolic and anabolic signaling pathways, and it also serves as a redox sensor and regulator, contributing to the preservation of normal cardiovascular function [152]. Resveratrol-induced activation of AMPK has been shown to effectively reduce superoxide production in mitochondria or NOX, concurrently promoting the expression of antioxidant genes like SOD and eNOS, thereby exerting suppressive effects on oxidative stress [152]. For instance, metformin, a drug used to treat hyperglycemia, triggers AMPK activation through the AMPK-PGC1α pathway in human umbilical vein endothelial cells (HUVECs), leading to reduced mitochondrial ROS levels, the induction of SOD2, and the enhancement of mitochondrial biogenesis, effectively countering hyperglycemia-induced oxidative stress [153]. Uncoupling protein 2 (UCP2) represents a critical mitochondrial antioxidant protein, and its deficiency has been associated with increased oxidative stress and inflammation, which disrupts blood flow and promotes AS plaque progression. Interestingly, overexpression of UCP2 has been shown to enhance AMPK phosphorylation in HUVECs, effectively inhibiting mitochondrial ROS production and attenuating the activity of the pro-inflammatory mediator forkhead box protein O1 (FoxO1). Consequently, the UCP2/AMPK pathway emerges as a pivotal regulator in maintaining homeostatic mitochondrial function, essential for mitigating oxidative stress and inflammation linked to AS development [154]. RSV exhibits protective effects against cardiovascular disease by activating various signaling pathways, including AMPK-SIRT1, AMPK-SIRT1-PGC-1α, AMPK-SIRT1-FoxOs, and AMPK-SIRT1-PPARα. Activation of these pathways leads to the upregulation of antioxidant enzymes and the inhibition of NF-κB, thereby attenuating oxidative stress [138]. Moreover, RSV demonstrates potent anti-inflammatory properties, which contribute to its cardioprotective effects by suppressing inflammation and oxidative stress. It achieves this by inhibiting NF-κB activation. Notably, several studies have reported that RSV activates the PI3K/Akt/mTOR signaling pathway while inhibiting NF-κB activation, activates the toll-like receptor 4(TLR4) signaling pathway while inhibiting NF-κB activation, and activates the Nrf2 signaling pathway while inhibiting NF-κB activation. These signaling pathways collectively exert significant inhibitory effects on the development of cardiovascular disease [139, 140, 155–157].

The PPAR plays a crucial role in regulating adipogenesis, and excessive lipid accumulation in adipose tissue is closely associated with an increased risk of cardiovascular disease [158]. RSV has been found to significantly enhance PPAR activity, particularly through the activation of the PPAR-γ/heme oxygenase 1(HO-1) signaling pathway. This activation leads to a reduction in the oxidative response of endothelial progenitor cells (EPCs) and promotes EPC re-endothelialization [159, 160]. Moreover, RSV has been shown to activate the expression of Krüppel-like Factor 2 (KLF2) in HUVECs, thereby providing protection against endothelial dysfunction. Activation of the SIRT1/KLF2 axis has been found to attenuate endothelial dysfunction by upregulating the expression of SIRT1 and subsequently increasing KLF2 levels [161]. Similarly, RSV-induced upregulation of KLF2 in EPCs has been shown to mitigate TNF-α-induced inflammatory injury and enhance EPC proliferation, adhesion, migration, angiogenesis, and nitric oxide (NO) bioavailability [162]. Furthermore, RSV indirectly targets the Nrf2 pathway to confer cardiovascular protection. Activation of Nrf2 by RSV reduces cardiac injury, improves cardiac function, protects endothelial function, and alleviates oxidative stress by enhancing Nrf2 activation, the Nrf2/ antioxidant response element (ARE) signaling pathway, the Nrf2/HO-1 signaling pathway, and Nrf2/ARE/HO-1 signaling [163, 164]. Importantly, all these targets have been demonstrated to stimulate eNOS production and expression, thereby increasing NO bioavailability and reducing oxidative stress [165].

### Curcumin

Curcumin is a lipophilic polyphenolic substance with an orange-yellow color derived from the rhizome of a herb. It has been recognized for its significant role in the prevention and treatment of cardiovascular disease, primarily attributed to its antioxidant and anti-inflammatory properties [166, 167]. Notably, curcumin has demonstrated anti-atherosclerotic effects, which can be ascribed to its ability to reduce elevated plasma cholesterol levels, inhibit LDL peroxidation, and attenuate lipid peroxidation [168]. The antioxidant properties of curcumin are implicated in modulating various signaling pathways associated with cardiovascular health. By virtue of its antioxidant activity, curcumin exerts protective effects against oxidative stress-induced damage, thereby mitigating the progression of cardiovascular disease. Additionally, curcumin possesses potent anti-inflammatory properties, which further contribute to its cardiovascular benefits. The modulation of inflammatory signaling pathways by curcumin helps attenuate the inflammatory response and subsequent tissue damage observed in cardiovascular pathologies.

Curcumin, a bioactive compound, exerts protective effects against OS in the cardiovascular system through multiple mechanisms. One of the key pathways involved is the activation of the SIRT1-FoxO1 pathway and the PI3K-Akt survival pathway, which leads to the attenuation of OS, reduction of ROS levels, and restoration of cardiac SOD levels [169]. Furthermore, curcumin demonstrates a capacity to mitigate mitochondrial oxidative damage induced by IR, thus reducing OS and improving postischemic cardiac function, myocardial infarct size, and myocardial apoptosis through the activation of SIRT1 signaling [170]. In experimental models of OS, curcumin exhibits inhibitory effects on the p53/p21 signaling pathway, resulting in a reduction of OS by suppressing p53 expression [171]. Curcumin's ability to enhance the resistance of cardiomyocytes to I/R damage and OS is attributed to the upregulation of Nrf2 signaling and its downstream target genes. Activation of the Nrf2/ARE pathways by curcumin restores MDA levels, enhances SOD activity, and protects myocardial morphology [172]. Moreover, curcumin induces the activity of cell protective enzymes, including SOD, through Nrf2 signaling activation in cerebellar granule neuron models [173]. Activation of the JAK/STAT pathway by curcumin has been demonstrated to confer protection against I/R myocardial injury in rats. Additionally, curcumin's modulation of the Nrf2/HO-1 pathway and inhibition of the janus kinase 2/ signal transducer and activator of transcription 3 (JAK2/STAT3) pathway contribute to the reduction of OS, inflammation, and myocardial fibrosis, thereby exerting beneficial effects in the prevention of cardiomyopathy [174, 175].

### Chlorogenic acid

Chlorogenic acid (CGA), a prominent phenolic compound found in green coffee extracts and tea, exhibits significant antioxidant activity and plays a crucial protective role in cardiovascular disease [176]. CGA exerts beneficial effects on endothelial barrier function and angiogenesis by activating the Rap1 signaling pathway and inhibiting the PI3K/Akt signaling pathway, thereby promoting the development of vascular endothelial cells [177]. Through the activation of SIRT1 signaling and the AMPK/PGC-1α pathway, CGA prevents oxidized low-density lipoprotein (oxLDL)-induced endothelial OS injury and mitochondrial dysfunction [178]. Furthermore, CGA has demonstrated notable efficacy in improving cardiac hypertrophy induced by ISO through the Akt/mammalian target of rapamycin (mTOR)/HIF-1α pathway. It also provides protection in patients with diabetic cardiomyopathy (DCM) by inhibiting glycosylation, modulating the PKCα- extracellular regulated protein kinases (ERK) signaling pathway, and exerting antioxidant effects [179, 180]. In experimental models, CGA has shown considerable reductions in pro-inflammatory cytokines, MI size, OS, and mitochondrial respiratory defects, while simultaneously enhancing antioxidant enzyme activity to protect the damaged myocardium [181, 182]. CGA exhibits protective effects against OS damage in endothelial cells exposed to hypochlorous acid, thereby improving vascular function. These effects are primarily attributed to the increased production of NO to prevent endothelial dysfunction and the induction of heme oxygenase-1 (Hmox-1) [183]. Similarly, CGA enhances NO formation in acidified saliva and reduces the intensity of free radicals [184].

### Salvianolic acid

Salvianolic acid, a natural polyphenolic compound derived from *Salvia miltiorrhiza*, exhibits significant protective effects against cardiovascular disease, primarily attributed to its antioxidant properties. Numerous studies have reported the ability of salvianolic acid to delay the development of ischemia by promoting angiogenesis, reducing infarct size, and improving post-infarction contractile function in animal models of MI [185, 186].

Salvianolic acid A (Sal A) pretreatment has demonstrated its ability to attenuate arsenic trioxide (ATO)-induced structural and functional damage to cardiac mitochondria. By reducing mitochondrial ROS overproduction, Sal A exerts a protective effect against ATO-induced cardiotoxicity in the heart. This protection is primarily attributed to the activation of the expression level of PGC-1α, which plays a crucial role in maintaining normal mitochondrial function [187]. Furthermore, Sal A has shown efficacy in preventing myocardial injury induced by lipotoxicity. By inhibiting the TLR4/ mitogen-activated protein kinase (MAPK) pathway, Sal A significantly improves palmitate-induced cardiomyocyte death while also restoring mitochondrial membrane potential and reducing intracellular ROS levels [188]. Sal A treatment has also been associated with the activation of signaling pathways that promote beneficial effects in adipose tissue and vascular protection. Activation of AMPK signaling and SIRT1 signaling contributes to increased white adipose tissue browning and reduced lipid accumulation [189]. In a mouse model of middle cerebral artery embolism (MCAO), Sal A induces neurovascular protection by inhibiting eNOS uncoupling and peroxynitrite production, while also upregulating phosphorylation of Akt, FoxO1, and ERK, thus protecting the brain from ischemia/reperfusion injury [190]. Moreover, Sal A shows promise in promoting renal tubular cell survival and ameliorating renal I/R injury through activation of the Akt/mTOR/4EBP1 pathway. This suggests that Sal A may be a potential candidate compound for the prevention of ischemic tissue injury in cardiovascular disease [191]. In experiments related to diabetes-associated macrovascular and renal injury, Sal A exhibits potential as an Nrf2 modulator with a protective effect on the vasculature. Its antioxidant effect is thought to be associated with reduced NF-κB activity and activation of the Nrf2/HO-1 pathway [192].

Salvianolic acid B (Sal B), one of the major active components derived from *Salvia miltiorrhiza*, exhibits inhibitory effects on the proliferation and migration of VSMCs. This inhibition is mediated by the induction of Nrf2 and HO-1 pathway expression. Sal B's strong antioxidant effects include reducing ROS production and modulating the NADP/NADPH ratio [193]. Moreover, Sal B demonstrates the ability to alleviate OS and improve endothelial dysfunction, thereby exerting significant protective effects against I/R injury in rats [194]. In addition, Sal B has been shown to attenuate acute myocardial ischemic injury induced by subcutaneous ISO administration in rats. This protective effect is achieved through the inhibition of intracellular ROS production, enhancement of mitochondrial membrane potential, and promotion of mitochondrial autophagy [195]. Furthermore, Sal B pretreatment has been found to provide substantial cardioprotection against cell death induced by ATO through the activation of the PI3K/Akt signaling pathway [196].

### Tea polyphenols

Tea is known to contain a rich array of biologically active compounds that confer beneficial effects on CVDs. Among these compounds, the polyphenols, particularly catechins and their derivatives such as catechin, epicatechin, and epigallocatechin gallate (EGCG), along with other polyphenols like gallic acid, chlorogenic acid, and various flavonoids, play a significant role [197]. The mechanisms underlying the preventive effects of tea polyphenols on CVDs involve multiple pathways. These include the reduction of blood lipid levels, improvement of I/R injury, attenuation of OS, enhancement of endothelial function, and protection of cardiomyocyte function [198].

The cardioprotective effects of catechins and their derivatives against I/R injury involve multiple molecular mechanisms, including modulation of the PI3K/Akt signaling pathway, c-Jun N-terminal kinase (JNK)/p38-MAPK pathway, preservation of mitochondrial function, and regulation of autophagy [199]. Specifically, in HUVECs, EGCG has been shown to inhibit eNOS uncoupling and mitigate endothelial dysfunction and apoptosis by activating the PI3K/Akt/eNOS pathway [200]. Moreover, EGCG can protect vascular endothelial cells from OS-induced injury by targeting the PI3K/Akt/mTOR pathway, thereby inducing autophagy [201]. Additionally, EGCG prevents endothelial dysfunction induced by ox-LDL in HUVECs through modulation of the Jagged-1/Notch signaling pathway [202]. In the context of I/R injury, EGCG has been reported to attenuate cardiomyocyte injury and modulate I/R-related molecules, leading to enhanced cell viability, reduced infarct size, and improved cardiac function recovery following ischemic injury [199]. EGCG activates the Nrf2/HO-1/NQO1 pathway in cardiac tissue, thereby reducing myocardial injury in mice with coronary heart disease. This activation of Nrf2 signaling by EGCG exerts antioxidant effects, improves blood lipid levels, and enhances SOD activity [203]. Furthermore, EGCG protects cardiomyocytes from I/R-induced injury by inhibiting signal transducer and activator of transcription 1(STAT1) activation, which is associated with hemodynamic recovery and improved ventricular function in rat hearts subjected to I/R [204].

### Honokiol

Magnoliae officinalis cortex, commonly referred to as "Houpo," is the dried bark of Magnolia officinalis and is widely utilized in traditional Chinese medicine [205]. This botanical resource is characterized by its abundance of bioactive compounds, including Honokiol (HKL). Notably, Honokiol has been recognized for its potential in conferring cardiovascular protection by virtue of its capability to scavenge free radicals and exert antioxidant effects within the body.

HKL is a naturally occurring biphenol compound that exhibits notable potential in blocking and ameliorating myocardial hypertrophy [206]. This effect is primarily attributed to HKL's ability to activate SIRT3, leading to increased levels and enhanced activity of this protein. Activated SIRT3, in turn, augments the antioxidant capacity of SOD and promotes the activation of PGC-1α. These actions collectively contribute to the reduction of ROS synthesis and mitigating OS within the heart. Furthermore, HKL demonstrates a protective role against OS-induced injury and apoptosis in a diabetic model of MI/R by activating the SIRT1-Nrf2 signaling pathway [207]. In studies involving HUVECs, HKL effectively inhibits palmitic acid (PA)-induced endothelial dysfunction. The underlying mechanism involves the suppression of pentraxin 3 expression, a marker of the inflammatory response, through inhibition of the IκB kinase (IKK)/IκB/NF-κB pathway. HKL achieves this by attenuating IκB phosphorylation and reducing the expression of NF-κB subunits (p50 and p65) [208]. Additionally, HKL demonstrates regulatory effects on iNOS, eNOS, and NO production, which collectively contribute to reducing endothelial cell injury and apoptosis [208]. Furthermore, HKL exerts modulatory effects on cardiac mitochondrial fatty acid respiration and atherosclerotic plaque formation. These effects are primarily mediated through the activation of AMPK and enhanced SOD activity [209, 210].

### Schisandra chinensis

The fruit extract of *Schisandra chinensis* (SC) and its bioactive lignan component exhibit notable therapeutic potential in the management of OS-related CVDs. Their beneficial effects encompass the activation of antioxidant defense systems, inhibition of pro-oxidant signaling pathways, and modulation of NO expression [211].

Treatment with schisandrol A, a bioactive component of SC, exhibited a protective effect in mice with acute MI. It significantly reduced infarct size, preserved cardiac function, and improved biochemical parameters and cardiac pathological changes. Mechanistically, schisandrol A was found to activate the PI3K/Akt pathway and inhibit the expression of NOX2 in H9c2 cells subjected to oxygen and glucose deprivation in acute MI mice, highlighting its potential therapeutic role in acute MI [212]. Another lignan found in SC, gomisin J, was shown to enhance the phosphorylation of eNOS and facilitate the translocation of eNOS in the cytoplasm of the rat thoracic aorta. This activation of the endothelium-dependent NO pathway and PI3K/Akt signaling resulted in increased NO production and subsequent relaxation of blood vessels [213]. Additionally, the dibenzocyclooctadiene lignan known as α-Iso-cubebene, present in SC, demonstrated the ability to inhibit high mobility group box-1 protein -induced monocyte to macrophage differentiation by suppressing ROS production in monocytes. This attenuation of vascular inflammation, coupled with endothelial proliferation associated with vascular injury, highlights the potential anti-inflammatory effects of α-Iso-cubebene [214]. SC extract itself exhibited antioxidant and cardioprotective effects in a rat model of isoproterenol-induced MI, possibly mediated through the Nrf2/HO-1 signaling pathway [215]. Moreover, schisandrin B and schisandrin C were found to attenuate endothelial defects induced by Ang II and improve aortic OS and vasorelaxation in mice by targeting the Kelch-like ECH-associated protein (Keap1)/Nrf2 pathway [216, 217]. Furthermore, schisandrin B exhibited potential in modulating OS-related cardiac insufficiency induced by DOX through the inhibition of MAPK/p53 signaling [218].

### Lignans of sesame

Sesame seeds and their bioactive lignan components, namely sesamin and sesamol, have been implicated in reducing the risk of cardiovascular disease by modulating OS and inflammation. Studies have shown that sesamol and sesamin effectively mitigate LPS-induced inflammation and OS factors in rats. Additionally, they prevent lipid peroxidation and restore SOD activity, thereby exerting protective effects [219]. Sesamin has demonstrated cardioprotective properties against DOX-induced cardiotoxicity and OS damage. It accomplishes this by activating the expression of Mn-SOD protein and stimulating SIRT1 activity [220]. Long-term sesamin treatment has been observed to improve arterial dysfunction in SHR by upregulating eNOS expression while downregulating p22 (phox) and p47 (phox) expression in NADPH oxidase [221]. Furthermore, sesamin promotes increased eNOS activity and enhances NO expression in ECs through the involvement of protein kinase A/CaMKII and calcium/calmodulin-dependent kinase β (CaMKKβ)/Akt/AMPK signaling pathways [222]. Sesamin also modulates the cardiac renin-angiotensin system in deoxycorticosterone acetate (DOCA)/salt rats, thereby ameliorating the development of left ventricular hypertrophy. This effect is achieved by inhibiting JNK/MAPK signaling expression and reducing OS injury [223]. Sesamol exhibits a protective effect against renal injury-associated atherosclerosis through the inhibition of the OS/IκB kinase α(IKKα)/p53 signaling pathway [224]. Furthermore, sesamin exerts its impact on reducing cardiovascular disease risk by inhibiting fatty acid synthesis and oxidation, cholesterol synthesis and absorption, and maintaining macrophage cholesterol homeostasis. These effects are mediated through the PPARα/PPARγ/liver X receptor α (LXRα) signaling pathways [225].

### Phillyrin and forsythin

Phillyrin and forsythin are bioactive constituents derived from the fruit of Forsythia suspensa, a medicinal plant. Phillyrin has demonstrated the ability to attenuate NE-induced cardiac hypertrophy both in vivo using C57BL/6 mice and in vitro using rat H9c2 cells. This effect is primarily achieved through the inhibition of the p38/ERK1/2 MAPK and Akt/NF-kB signaling pathways. Moreover, phillyrin pretreatment has been shown to significantly improve cardiac function, reduce ROS production, and mitigate the inflammatory response [226]. Forsythin, on the other hand, has been found to suppress LPS-induced inflammation in RAW 264.7 mouse monocyte macrophage leukemia cells. This anti-inflammatory activity is attributed to its ability to inhibit JAK-STAT and p38 MAPK signaling pathways, as well as reducing ROS generation [227].

### Cinnamic acid

Cinnamic acid (CA) is an organic acid obtained from cinnamon bark or benzoin and is known for its diverse range of biological activities, including antioxidant and anti-inflammatory properties. Notably, CA has been found to exhibit inhibitory effects on PDGF-BB-induced proliferation of VSMCs. This inhibition is achieved through the upregulation of p21 and p27 protein expression levels, both of which are key regulators of cell cycle progression and proliferation [228].

CA and cinnamaldehyde have demonstrated protective effects against ischemic myocardial injury induced by ISO in a rat model. This protection is attributed to their ability to enhance the anti-OS effect of NO and increase SOD activity in cardiac tissue [229]. Recent research indicates that CA plays a role in attenuating ischemic heart disease by inhibiting the NLRP3/cysteinyl aspartate specific proteinase-1(Caspase-1)/gasdermin D (GSDMD) signaling pathway. Moreover, CA pretreatment significantly improves cardiac structural function, reduces myocardial infarct size and injury, and diminishes OS and inflammatory responses in a rat model of MI/RI [230]. Additionally, both CA and cinnamyl alcohol exhibit vasodilatory effects by activating the NO-cGMP-PKG pathway, while also inhibiting Rho-kinase [231, 232].

Ferulic acid (FA), a derivative of cinnamic acid, is an important active ingredient found in various Chinese medicines, including the root of *Angelica sinensis* (Oliv.) Diels. FA exhibits notable protective effects, particularly against OS, inflammation, vascular endothelial damage, and platelet aggregation associated with CVDs. One of its mechanisms involves the inhibition of the PI3K/Akt pathway, ROS production, and aldose reductase activity, thereby protecting the vascular endothelium through ERK1/2 and NO/ET-1 signaling pathways [233]. Furthermore, FA has been shown to suppress H2O2-induced inflammation and OS in rat VSMCs by inhibiting the MAPK/Akt and NF-κB pathways [234]. In a study investigating the protective effect of FA against DOX-induced cardiotoxicity, it was observed that FA could reduce excessive ROS production and NADPH oxidase activation by activating the Nrf-2/HO-1 signaling pathway and the PI3K/Akt/mTOR signaling pathway while inhibiting MAPK activation and the NF-κB pathway. This ultimately mitigated OS in cardiac myocytes [235]. Additionally, during I/R injury, FA demonstrated its ability to attenuate myocardial oxidative damage by inhibiting SDH. This inhibition led to a reduction in succinate levels, thereby mitigating excessive intracellular ROS production and OS [236].

### Syringic acid

Syringic acid (SA) is a naturally occurring compound synthesized in plants through the shikimate pathway. It can be sourced from various plants, including *Conyza canadensis* (L.) Cronq in the Asteraceae family and *Rhododendron dauricum* L. in the Rhododendron family. The shikimate pathway serves as the primary route for SA biosynthesis in plants.

SA exhibits cardioprotective effects in a rat model of MI induced by ISO. These effects may be attributed to its ability to counteract lipid peroxidation and enhance endogenous antioxidant systems, such as increasing the content of reduced GSH [237]. Similarly, syringaldehyde, another compound with antioxidant and anti-inflammatory properties, also demonstrated cardioprotective effects in the ISO-induced MI model [238]. SA was found to activate the PI3K/Akt/GSK-3β signaling pathway, leading to increased levels of phosphorylated PI3K, Akt, GSK-3β, and mitochondrial Cyt c. This activation provided a protective effect on the hearts of rats subjected to I/R injury [239]. Furthermore, the combination of SA and RSV (referred to as combination) exhibited cardioprotective abilities against ISO-induced cardiotoxicity in rats. This effect was achieved by inhibiting the NF-kB and TNF-α pathways, suppressing lipid peroxidation, reducing NF-kB and TNF-α mRNA expression, and increasing the activities of SOD and CAT [240].

### Oilanolic acid

Oleanolic acid (OA) is a pentacyclic triterpenoid that is widely present in Chinese medicine. It can be isolated and extracted from various sources, including the whole herb of Swertia plants in the Gentianaceae family or the fruit of *Ligustrum lucidum*. OA exhibits beneficial effects on cardiovascular protection, including hypolipidemic, anti-AS activity, and hypotensive effects [241, 242]. The cardioprotective properties of OA involve the activation of the Nrf2/HO-1 signaling pathway, which helps mitigate ECs damage and reduce OS [243]. In HUVECs, OA prevents OS-induced apoptosis by activating the Akt/eNOS signaling pathway, thereby enhancing eNOS activity and reducing ROS levels. OA also attenuates mitochondrial damage, restores NO production, and enhances the activities of SOD and CAT [244]. Furthermore, OA demonstrates its ability to attenuate cardiac remodeling during aging by modulating mitochondrial autophagy and ameliorating mitochondrial ultrastructural abnormalities [245].

## Herbal monomers with powerful antioxidant capacity: flavonoids

### Baicalin

Baicalin (BA) is a bioactive flavonoid that can be isolated from the roots of the traditional herb *Scutellaria baicalensis* Georgi. It constitutes approximately 10.11% of the content in the roots of *Scutellaria baicalensis* Georgi. BA has been extensively studied for its antioxidant and anti-inflammatory properties, particularly in the context of cardiovascular disease disorders [246, 247]. The cardioprotective effects of BA are attributed to its ability to attenuate NF-κB activity and inhibit inflammatory cell infiltration. Additionally, BA exerts its beneficial effects by suppressing the production of pro-inflammatory cytokine TNF-α, scavenging ROS, and enhancing the endogenous antioxidant capacity [248].

BA, a bioactive flavonoid derived from the roots of *Scutellaria baicalensis* Georgi, exhibits diverse effects on cardiovascular health. It demonstrates antiproliferative and migratory properties in VSMCs and effectively attenuates carotid artery neointimal hyperplasia. These effects may be mediated, at least in part, by the upregulation of smooth muscle 22 alpha (SM22α) expression, suppression of ROS production, and inhibition of ERK phosphorylation [249]. Furthermore, BA mitigates hypoxia-induced pulmonary vascular remodeling and exerts an antiproliferative effect on PASMCs. This action is associated with a reduction in HIF-α expression and inhibition of the PI3K/Akt pathway. Notably, BA restores cell cycle regulation and upregulates the cell cycle protein-dependent kinase inhibitor p27, likely through the Akt/HIF-1α-related signaling pathway [250]. Another study highlights the ability of BA to alleviate chronic hypoxia-induced PAH by enhancing adenosine A2A receptor activity and suppressing stromal cell-derived factor-1 (SDF-1)/C-X-C chemokine receptor type 4 (CXCR4)-induced PI3K/Akt signaling [251]. BA also attenuates Ang II-induced endothelial dysfunction and oxidative stress by promoting endothelial vasodilation, inhibiting apoptosis of HUVECs, and enhancing antioxidant capacity. These effects are associated with the upregulation of the PI3K/Akt/eNOS pathway [252]. In the context of hyperglycemia-induced cardiovascular malformations, BA administration effectively inhibits ROS and induces autophagy, thereby mitigating developmental abnormalities during early chick embryonic development [253]. Moreover, BA treatment protects against ATO-induced cardiotoxicity by suppressing oxidative stress, inflammatory factor expression, and by enhancing endogenous antioxidant systems through the inhibition of the TLR4/NF-κB signaling pathway [254]. BA significantly improves cardiac function, reduces MI size, inhibits cardiomyocyte apoptosis, and alleviates myocardial I/R injury, particularly in cardiac microvascular endothelial cells. These cardioprotective effects are attributed to the activation of the PI3K-Akt-eNOS signaling pathway and the promotion of NO production in cardiac microvascular endothelial cells of rats with myocardial I/R injury [255]. Additionally, BA treatment effectively reduces atherosclerotic lesion size and lipid accumulation in carotid arteries of AS rabbits. This effect is mediated through the PPARγ-LXRα signaling pathway, which enhances the expression of ATP-binding cassette transporter A1 (ABCA1) and ABCG1. Hence, BA exerts anti-atherosclerotic effects via the PPARγ-LXRα-ABCA1/ABCG1 pathway [256]. Lastly, BA activates brown adipose tissue and white adipose tissue through the activation of AMPK/PGC1α signaling, thereby reducing the risk of cardiovascular disease [257].

### Quercetin

Quercetin, a widely distributed flavonoid in plants, exhibits multiple mechanisms that contribute to its potential in reducing the risk of OS-related CVDs. It exerts its effects through various pathways, including the reduction of ox-LDL levels, inhibition of OS, mitigation of endothelial dysfunction, protection of endothelial function, suppression of adhesion molecules and inflammatory markers, and inhibition of platelet aggregation [258].

The administration of quercetin has been shown to exhibit beneficial effects on endothelial dysfunction and AS in various experimental models. In SHR rats, quercetin treatment suppressed endothelial dysfunction by downregulating NADPH oxidase activity in VSMCs and enhancing eNOS activity [259, 260]. In mice fed a HFD, quercetin demonstrated anti-endothelial dysfunction and AS effects, which were mediated by increased HO-1 protein expression and improved NO bioavailability [261]. Moreover, long-term application of quercetin activated the Nrf2/HO-1 signaling pathway and enhanced the stability of HIF1α in HUVECs, thereby reducing endothelial dysfunction and AS [262]. Additionally, quercetin exhibited protective effects against iron overload damage in HUVECs through the activation of the ROS/asymmetric dimethylarginine (ADMA)/dimethylarginine dimethylaminohydrolase II (DDAHII)/eNOS/NO pathway. This was evidenced by the reduction in ROS production, increased expression and activity of DDAHII leading to decreased ADMA levels, and subsequent inhibition of eNOS uncoupling, ultimately resulting in increased NO content [263].

Quercetin exerts protective effects on the heart through various signaling pathways. In cardiomyocytes subjected to hypoxia/reoxygenation (H/R)-induced injury, quercetin attenuates mitochondrial autophagy and inhibits ER stress and OS injury by activating the SIRT1/transmembrane BAX inhibitor-1 motif-containing 6(TMBIM6) signaling pathway, thereby reducing damage [264]. Quercetin also activates SIRT5, leading to the desuccinylation of IDH2, which helps maintain mitochondrial homeostasis, attenuate inflammatory responses and OS damage, and improve cardiac function in a mouse model of myocardial fibrosis and heart failure induced by transverse aortic constriction (TAC) [265]. Furthermore, quercetin inhibits OS and promotes mitochondrial homeostasis, thereby reducing VSMCs apoptosis and mitigating vascular calcification [266]. The protective effect of quercetin against ISO-induced myocardial ischemia may also involve the inhibition of calcium influx [267]. In attenuating I/R injury, the cardioprotective effects of quercetin may be associated with the activation of the SIRT1/PGC-1α signaling pathway, JAK2/STAT3 signaling, and PI3K/Akt kinase pathways [268, 269].

### Luteolin

Lignans, derived from traditional Chinese herbs, have been recognized for their potential cardioprotective effects mediated through multiple signaling pathways. These compounds exhibit antioxidant and anti-inflammatory properties and have shown the ability to alleviate endothelial dysfunction. The mechanisms underlying the cardioprotective effects of lignans involve their antioxidant activity and modulation of inflammatory responses. Furthermore, lignans have been found to improve endothelial function.

Lignans, derived from traditional Chinese herbs, have emerged as potential cardioprotective agents through their modulation of various signaling pathways. Lignocaine, a specific lignan compound, has been shown to mitigate OS damage and inflammatory factors in HUVECs by inhibiting the STAT3 pathway. This inhibition results in reduced ROS production and inhibition of STAT3 activation, leading to a decrease in the production of oxysterols and hydroxylated fatty acids, which are implicated in the pathogenesis of atherosclerosis and other CVDs [270]. Lignans also play a crucial role in mitigating the proliferation and apoptosis of VSMCs in atherosclerosis. They achieve this by inhibiting NADPH oxidase activity and ROS production in HUVECs through activation of the AMPK/PKC pathway [271]. Another lignan compound, luteolin, has demonstrated the ability to attenuate OS and inflammatory responses in HUVECs induced by TNF-α). This attenuation is achieved by inhibiting the NOX4/ROS/NF-κB signaling pathways, resulting in reduced expression of NOX4 and NF-κB and enhanced endogenous antioxidant capacity, such as SOD and GSH [272]. Furthermore, luteolin protects HUVECs from iron overload-induced damage by activating the ROS/ADMA/DDAHII/eNOS/NO pathway, preserving normal mitochondrial function, and reducing OS damage. This pathway holds promise as a potential target for cardiac protection [273]. Lignans have also been found to alleviate inflammatory phenotype and OS induced by high glucose (HG) in various cellular models. These effects are mediated through inhibition of the NF-κB pathway and activation of the Nrf2 signaling pathway, highlighting the potential of lignans in preventing diabetic cardiovascular complications [274]. Moreover, lignans exhibit protective effects against cardiac fibrosis, hypertrophy, and dysfunction induced by streptozotocin in mice by modulating these pathways [275]. The activation of the kidney injury marker 1 (Kim-1)/NF-κB/Nrf2 signaling pathway by lignocaine has been shown to protect against sodium fluoride-induced hypertension and associated cardiovascular complications, leading to improved NO bioavailability [276].

In the context of I/R injury, lignocaine has been shown to exert cardioprotective effects by reducing the expression of SHP-1 and promoting phosphorylation of STAT3 in rat heart tissue and H9c2 cells [277]. Another important protein involved in the regulation of oxidative stress is Sestrin2, which plays a role in activating the antioxidant signaling pathway mediated by Nrf2. Lignans have been found to enhance the transcription of Sestrin2, thereby promoting its interaction with Nrf2 and facilitating antioxidant effects to mitigate mitochondrial damage and alleviate myocardial I/R injury in the context of diabetes [278].

In a rat model of hypoxic pulmonary hypertension (HPH), luteolin has been demonstrated to reduce pulmonary vascular remodeling and improve pulmonary vascular endothelial cell function. These effects are mediated through the hypoxia-inducible factor-2 alpha (HIF-2α)/NO axis and the PI3K/Akt/eNOS signaling pathway. Luteolin treatment leads to a reduction in hypoxia-induced HIF-2α expression, which subsequently promotes the expression of nitric oxide (NO). Additionally, luteolin treatment enhances the expression of endothelial nitric oxide synthase (eNOS) without increasing its activity. The activation of the PI3K/Akt signaling pathway by luteolin contributes to the increased bioavailability of NO by regulating the activity of eNOS, as PI3K/Akt is known to be an upstream regulator of eNOS. These findings suggest that luteolin exerts its beneficial effects on HPH by modulating the HIF-2α/NO axis and the PI3K/Akt/eNOS signaling pathway [279].

In a rat model of hypoxic pulmonary hypertension (HPH), luteolin has been demonstrated to reduce pulmonary vascular remodeling and improve pulmonary vascular endothelial cell function. These effects are mediated through the HIF-2α/NO axis and the PI3K/Akt/eNOS signaling pathway. Luteolin treatment leads to a reduction in hypoxia-induced HIF-2α expression, which subsequently promotes the expression of NO. Additionally, luteolin treatment enhances the expression of eNOS without increasing its activity. The activation of the PI3K/Akt signaling pathway by luteolin contributes to the increased bioavailability of NO by regulating the activity of eNOS, as PI3K/Akt is known to be an upstream regulator of eNOS. These findings suggest that luteolin exerts its beneficial effects on HPH by modulating the HIF-2α/NO axis and the PI3K/Akt/eNOS signaling pathway [279]. In contrast, another study investigating the effects of luteolin on monocrotaline (MCT)-induced PAH in rats revealed different findings. In this experiment, inhibition of the PI3K/Akt signaling pathway was shown to improve pulmonary vascular remodeling, as well as reduce the proliferation and migration of PASMCs. These observations suggest that the role of the PI3K/Akt signaling pathway may vary depending on the specific pathological context, such as HPH or MCT-induced PAH [280].

### Naringin

Naringin, a natural flavonoid found in various herbs, exhibits protective effects against OS-induced heart damage and cardiovascular dysfunction. Its antioxidant activity enables scavenging of free radicals and mitigating OS-related damage [281]. In HUVECs, naringin attenuates autophagy by inhibiting the activation of the PI3K-Akt-mTOR signaling pathway, thereby alleviating dysfunction induced by HG and HFD conditions [282]. Furthermore, naringin improves mitochondrial and cardiac dysfunction induced by HFD, reduces blood lipid concentrations, and mitigates OS [282]. Activation of the HIF-1α/ Bcl2 interacting protein 3(BNIP3) signaling pathway by naringin protects against hypoxia/ischemia-induced myocardial injury by enhancing autophagic flux [283]. Naringin preconditioning reverses HG-induced myocardial damage by inhibiting the MAPK pathway, suppressing the NF-κB pathway, upregulating ATP-sensitive K(+) channels, and restoring SOD levels [284]. Modulation of the leptin-JAK2/STAT3 pathway in H9c2 cardiac cells by naringin ameliorates myocardial injury [285]. In hypercholesterolemic rats, naringin treatment attenuates vascular OS and endothelial dysfunction by reducing the protein expression of lipoprotein receptor-1, NADPH oxidase subunits, and iNOS [286]. Naringenin treatment demonstrates significant reduction of sodium arsenite-induced cardiotoxicity in rats. Naringenin effectively prevents abnormal Na–K-ATPase activity induced by arsenic toxicity, potentially through its membrane stabilizing properties. Naringenin also upregulates the expression levels of Nrf-2 and HO-1, increases myocardial mitochondrial enzyme activity, and improves the structural morphology of the heart [287].

## Other types of herbal monomers with antioxidant activity

Alkaloids are a class of nitrogenous organic compounds characterized by complex cyclic structures and basic properties. They are widely distributed in dicotyledonous plants. One notable alkaloid is berberine (BBR), which is isolated from the Chinese herb Huanglian and serves as the main active ingredient. Studies have demonstrated the significant role of BBR in post-MI myocardial cell injury and its ability to improve ventricular remodeling and OS injury in a mouse model of myocardial ischemia. This effect is achieved through modulation of the Wnt5a/β-catenin signaling pathway [288]. BBR also downregulates myocardial PERK and eIF2α phosphorylation, inhibits activating transcription factor 4 and C/EBP-homologous protein expression, and activates the JAK2/STAT3 signaling pathway, leading to attenuation of ER stress-induced apoptosis and improvement of MI/R injury in rats [289]. Furthermore, BBR mediates SIRT1 to inhibit the expression of p66Shc and enhance the activity of CAT, SOD, and GSH-PX. This mechanism reduces MDA levels and ameliorates doxorubicin-induced cardiomyopathy in rats [290]. BBR can also mediate the Klotho/SIRT1 signaling pathway to mitigate mitochondrial dysfunction and reverse aging-related heart injury. By upregulating Klotho expression and downregulating SIRT1 expression, BBR exerts antioxidant effects and prevents cardiac aging [291]. Another alkaloid, sinomenine (SIN), extracted from Caulis sinomenii, exhibits cardioprotective effects by inhibiting the reduction in B-cell lymphoma 2 (Bcl2) levels and the increase in Caspase-3, Caspase-9, and Bcl2-Associated x(Bax) expression in ischemic myocardial tissues following MI/R injury. This effect is attributed to modulation of the OS pathway, leading to a significant reduction in myocardial infarct size and myocardial injury markers in MI/R-injured mice [292]. SIN also improves antioxidant parameters, such as MDA, SOD, CAT, and GPX levels, and inhibits the levels of CK, CK-MB, and troponin I in MI/R-injured rats, thereby exerting anti-arrhythmic effects against OS volatiles [293]. Studies further demonstrate that SIN reduces apoptosis rates, ROS levels, and MDA expression by activating the Nrf2/ARE signaling pathway, thereby attenuating OS associated with cardiac hypertrophy [294].

Saponins, a diverse group of compounds, serve as the principal bioactive components in numerous Chinese medicines, exhibiting various pharmacological activities, including antioxidative stress effects. Astragaloside IV (AS-IV), a prominent active component derived from Astragalus, exerts inhibition on the ROS/Caspase-1/GSDMD signaling pathway, leading to attenuation of myocardial fibrosis and cardiac remodeling induced by MI [295]. Moreover, AS-IV prevents ROS generation resulting from succinate accumulation, promotes Nrf2 nuclear translocation for ROS scavenging, and counteracts lipid peroxidation triggered by excessive ROS, thereby mitigating MI/R injury [296]. AS-IV also activates the Nrf2/HO-1 signaling pathway, thereby improving myocardial hypertrophy in chronic heart failure (CHF) rats [297]. Additionally, studies have demonstrated that AS-IV significantly reduces DOX-induced cardiomyocyte death, apoptosis, and cardiac insufficiency by inhibiting NOX2- and NOX4-mediated oxidative stress [298]. Ginsenoside, the major active constituent of ginseng, possesses diverse pharmacological effects, including antioxidant properties. Ginsenoside Rb1 regulates glucose and lipid metabolism through an adipocytokine-mediated pathway and enhances basal GSH reductase activity and GSH levels, thereby reducing ROS levels and attenuating oxidative stress, excessive lipid accumulation, cardiac hypertrophy, and apoptosis in the myocardium of hyperglycemic/hyperlipidemic diabetic mice [299, 300]. Ginsenoside Rb3 mitigates oxidative stress, elevates total antioxidant levels, and reduces myocardial infarct size in MI/R-injured rats in both in vitro and in vivo settings by activating the PERK/Nrf2/Hmox1 signaling pathway [300]. Additionally, ginsenoside Rg1 inhibits caspase-3 expression, restores Bcl-xL expression, alleviates oxidative stress, and protects against myocardial injury in diabetic rats [301]. Moreover, ginsenoside Rg3 counteracts angiotensin II-induced myocardial hypertrophy by modulating the SIRT1/NF-κB pathway, leading to inhibition of NLRP3 inflammasome expression and improvement of oxidative stress [302].

Quinones, primarily sourced from natural plants belonging to the Rubiaceae, Polygonaceae, Leguminosae, Rhamnaceae, and Liliaceae families, exhibit a diverse array of pharmacological effects. Tanshinone IIA, the principal active constituent of tanshin, exerts notable properties, including the reduction of radiation-induced ROS production in cardiomyocytes, upregulation of SOD levels, modulation of the p38/p53 MAPK signaling pathway, attenuation of radiation-induced cardiomyocyte apoptosis, and improvement of radiation-induced myocardial injury [303]. Treatment with Tanshinone IIA reduces PERK and eukaryotic translation initiation factor 2α expression in cardiomyocytes, thereby ameliorating ER stress, reducing cardiomyocyte apoptosis, mitigating ROS production, and attenuating oxidative stress, consequently inhibiting MI and enhancing myocardial function [304]. Furthermore, Tanshinone IIA safeguards cardiomyocytes against oxidative stress-induced injury and apoptosis by enhancing scavenging of oxygen free radicals, preventing lipid peroxidation, and upregulating the Bcl-2/Bax ratio [305].

**Fig. 4:**
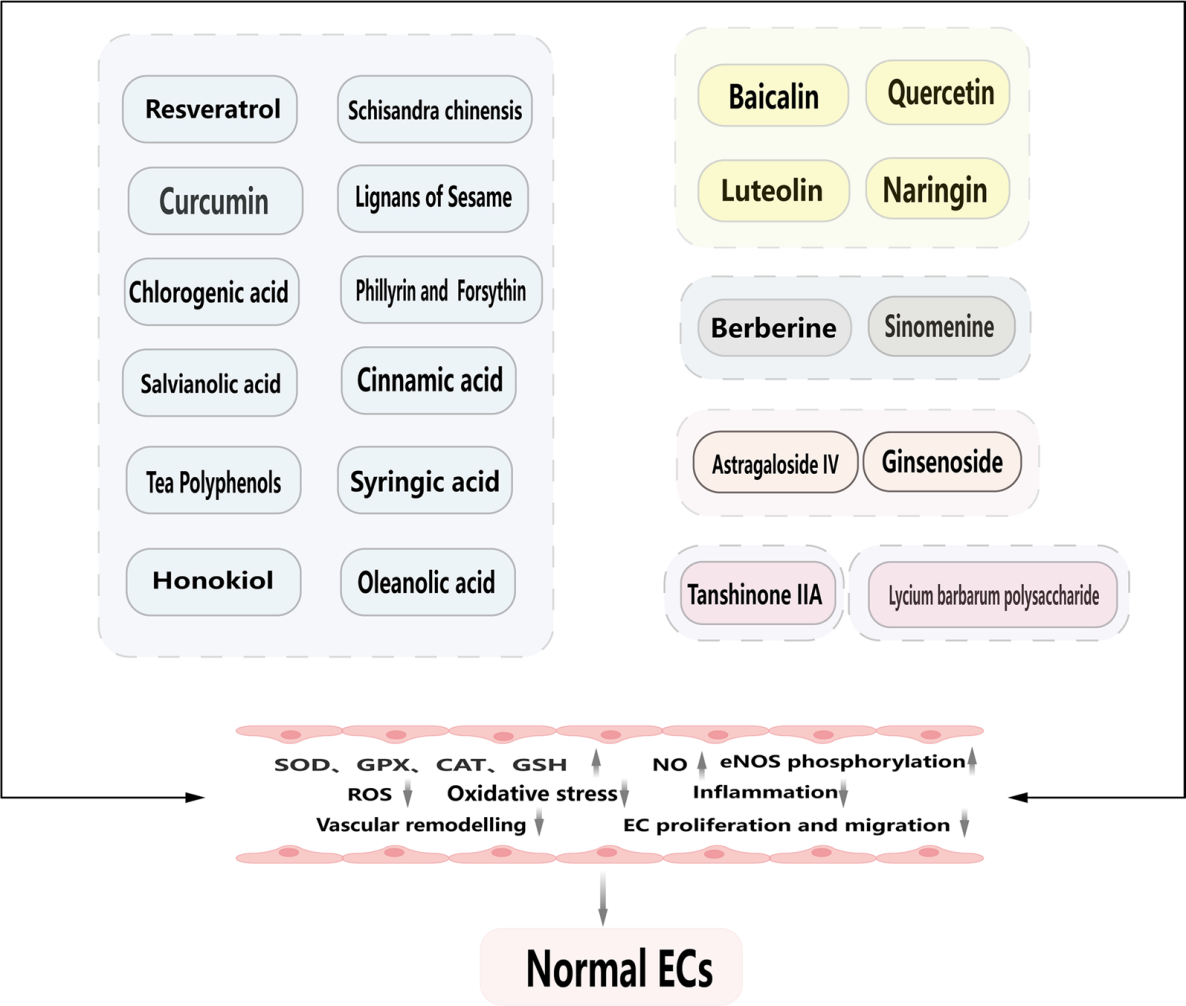
Herbal monomers such as polyphenols, flavonoids, alkaloids, saponins, quinones and polysaccharides inhibit the expression of ROS through various signaling pathways and reduce the damage caused by OS to the organism. This can be reflected by the upregulation of SOD, GPX, CAT, GSH, NO, eNOS phosphorylation and downregulation of inflammation, ROS, vascular remodeling, ECs proliferation and migration

Polysaccharides, which are widely distributed in animals, plants, and microorganisms, exhibit diverse biological activities, including antioxidant and immunomodulatory effects. Among them, *Lycium barbarum* polysaccharide (LBP) serves as the principal bioactive component in *Lycium barbarum* and contributes significantly to its medicinal properties, particularly its antioxidant activity. In the context of MI/R injury, LBP has been shown to downregulate the expression of G protein-coupled receptor kinase 2 in rats, thereby restricting myocardial infarct size through activation of the Akt/eNOS signaling pathway. Moreover, LBP demonstrates anti-apoptotic effects and mitigates oxidative stress [306]. Notably, LBP administration in a rat model of heart failure resulted in a significant reduction in plasma lipid peroxidation levels, as indicated by decreased MDA content [307]. Furthermore, LBP supplementation exhibited a noteworthy impact on ameliorating DOX-induced acute cardiotoxicity by increasing the activities of SOD and GSH-Px and reducing myocardial MDA levels [308] (Fig. 4; Table 1).

**Table 1:** Herbal monomers alleviate OS-related cardiovascular disease by activating or inhibiting different signaling pathways

Drugs	Experimental models	Signaling pathways and related mechanisms	References
Resveratrol	Clinical trial	inhibition of oxLDL	[143]
	MTHFR C677T vector subjects and mice	Activation of SIRT1/FoxO	[145]
	SIRT1flox/flox mice	Activation of SIRT1 and inhibition of NLRP3	[147]
	H/R-induced rat cardiomyocytes	Activation of SIRT1/SIRT3/Mfn2/Parkin/PGC-1α	[148]
	PH Murine Model	Activation of SIRT1/SIRT3	[149]
	3T3-L1 cells	Activation of AMPK/SIRT	[151]
	HUVECs	Activation of UCP2/AMPK	[154]
	C57BL/6 mice	Activation of AMPK/SIRT/PGC-1α	[138]
	SD rat Model	Activation of PI3K/Akt/mTOR and inhibition of NF-κB	[139]
	HUVECs	Inhibition of NF-κB	[155]
	I/R rats	Inhibition of TLR4/NF-κB	[156]
	SD rats	Activation of SIRT1 and inhibition of NF-κB	[157]
	H9C2 cells	Activation of Nrf2 and inhibition of NF-κB	[140]
	EPCs	Activation of PPAR-γ/HO-1	[160]
	HUVECs	Activation of SIRT1/KLF2	[161]
	MI/R rats and ECs	Activation of Nrf2/ARE	[163, 164]
Curcumin	DCM rats	Activation of SIRT1/FoxO1 and PI3K/Akt	[169]
	I/R rats	Activation of SIRT1	[170]
	D-gal induced rats	Inhibition of P53/p21	[171]
	DCM rats	Activation of Nrf2/ARE	[172]
	MI/R rats	Activation of JAK2/STAT3	[175]
Chlorogenic acid	HUVECs	Activation of Rap1 signaling and inhibition of PI3K/Akt	[177]
	HUVECs	Activation of SIRT1/AMPK/PGC-1 and inhibition of oxLDL	[178]
	ISO induced rats	Activation of Akt/mTOR/HIF-1α	[179]
	H9C2 cells	Inhibition of PKCα-ERK	[180]
	HAEC	Activation of Hmox-1	[183]
Salvianolic acid	H9C2 cells	Activation of PGC-1α	[187]
	H9C2 cells	Inhibition of TLR4/MAPK	[188]
	HFD induced mice	Activation of AMPK/SIRT1	[189]
	MCAO	upregulating Akt/FoxO1/ERK	[190]
	I/R-injured rats	Activation of Akt/mTOR/4EBP1	[191]
	Diabetic rats	Activation of Nrf2/ARE and inhibition of NF-κB	[192]
Tea Polyphenols	H9C2 cells	Activation of PI3K/Akt	[199]
	H9C2 cells	Activation of JNK/p38-MAPK	[199]
	HUVECs	Activation of PI3K/Akt/eNOS	[200]
	HUVECs	Activation of PI3K/Akt/mTOR	[201]
	HUVECs	inhibition of oxLDL	[202]
	CHD mice	Activation of Nrf2/HO-1/NQO1	[203]
	I/R-injured rats	Inhibition of STAT1	[204]
Honokiol	SIRT3 KO mice	Activation of SIRT3/ PGC-1α	[206]
	MI / R injured rats	Activation of SIRT1/Nrf2	[207]
	HUVECs	Inhibition of IKK/IκB/NF-κB	[208]
	T2DM mice	Activation of AMPK	[209]
*Schisandra chinensis*	H9c2 cells	Activation of PI3K/Akt	[212]
	HCAECs	Activation of eNOS/PI3K/Akt	[213]
	THP-1 cells	Inhibition of ROS	[214]
	MI rats	Activation of Nrf-2/HO-1	[215]
	RAECs	Activation of Keap1/Nrf2	[216, 217]
	DOX-induced mice	Inhibition of MAPK/P53	[218]
Lignans of Sesame	DOX-induced mice	Activation of SIRT1	[220]
	SHR rats	Up-regulation eNOS and down-regulation p22/p47	[221]
	HUVECs	Activation of CaMKKβ/Akt/AMPK	[222]
	DOCA/salt rats	Inhibition of JNK/MAPK	[223]
	HAECs	Inhibition of OS/IKKα/p53	[224]
	CHO cells	Activation of PPARα/PPARγ/LXRα	[225]
Phillyrin and Forsythin	H9c2 cells	Inhibition of p38/ERK1/2MAPK and Akt/NF-kB	[226]
	RAW264.7	Inhibition of JAK-STA T/p38MAPK	[227]
Cinnamic acid	IHD rats	Up-regulation SOD and NO of expression	[229]
	MI / RI rats	Inhibition of NLRP3/Caspase1/GSDMD	[230]
	HUVECs	Activation of NO/cGMP/PKG and inhibition of Rho-kinase	[231, 232]
	VSMCs in rats	Inhibition of MAPK/Akt pathways and NF-κB	[234]
	DOX-induced rats	Activation of Nrf-2/HO-1, PI3K/Akt/mTOR and inhibition of MAPK/NF-κB	[235]
	H9c2 cells	Inhibition of SDH	[236]
Syringic acid	MI rats	Up-regulated antioxidant capacity and anti-lipid peroxidation	[237, 238]
	MIRI rats	Activation of PI3K/Akt/GSK-3β	[239]
	MI rats	Inhibition of NF-kB and TNF-α	[240]
Oleanolic acid	HUVECs	Activation of Nrf2/HO-1	[243]
	HUVECs	Activation of Akt/eNOS	[244]
Baicalin	Arterial ligation mice	Upregulation of SM22α expression and inhibition ERK	[249]
	PASMCs	Inhibition of PI3K/Akt/HIF-1α and upregulation of P27 expression	[250]
	A2AR-deficient nice	Upregulation of SDF-1/CXCR4 expression and inhibition PI3K/Akt	[251]
	HUVECs	Activation of PI3K/Akt/eNOS	[252]
	ATO-induced mice	Inhibition of TLR4/NF-κB	[254]
	CMECs	Activation of PI3K/Akt/ eNOS	[255]
	AS rabbit	Activation of PPARγ/LXRα	[256]
Quercetin	AS mice	Upregulation of HO-1 expression	[261]
	HUVECs	Activation of Nrf2/HO-1 and upregulation of HO-1 expression	[262]
	HUVECs	Activation of ADMA/DDAHII	[263]
	Human cardiomyocytes	Activation of SIRT1/TMBIM6	[264]
	TAC mice	Activation of SIRT5	[265]
	MIRI rats	Activation of SIRT1/PGC-1α	[268]
	MIRI rats	Activation of JAK2/STAT3	[269]
Luteolin	HUVECs	Inhibition of STAT3	[270]
	HUVECs	Activation of AMPK/PKC	[271]
	HUVECs	Inhibition of NOX4/ROS/NF-κB	[272]
	HUVECs	Activation of ADMA/DDAHII	[273]
	Diabetic Rat	Activation of Keap1/Nrf2	[274]
	H9C2 cells	Activation of Nrf2 and inhibition of NF-κB	[275]
	ISO-induced mice	Activation of Kim-1/NF-kB/Nrf2	[276]
	Activation	Activation of STAT3	[277]
	Diabetic rats	Activation of Sestrin2 and Nrf2	[278]
	HPH rats	Activation of PI3K/Akt and inhibition of HIF-2α	[279]
	MCT rats	Inhibition of PI3K/Akt	[280]
Naringin	HUVECs	Activation of PI3K/Akt/mTOR	[282]
	H9C2 cells	Activation of HIF‑1α/BNIP3	[283]
	H9C2 cells	Inhibition of MAPK, NF-kB and upregulation of ATP-sensitive K(+)	[284]
	H9C2 cells	Activation of leptin-JAK2/STAT3	[285]
	Arsenic toxicity rats	Activation of Nrf2/HO-1	[287]
Berberine	MI mice	Inhibition of Wnt5a/β-catenin	[288]
	MI rats	Activation of JAK2/STAT3	[289]
	H9C2 cells	Activation of SIRT1	[290]
	H9C2 cells	Activation of Klotho/SIRTI	[291]
Sinomenine	MIRI mice	Inhibition of Bcl2 expression and the increase in caspase-3, caspase-9 and Bax expression	[292]
	I/R rats	Activation of endogenous antioxidant capacity and inhibition of inflammatory factors	[293]
	H9C2 cells	Activation of Nrf2/ARE	[294]
Astragaloside IV	MI mice	Inhibition of ROS/Caspase-1/GSDMD	[295]
	I/R rats	Activation of Nrf2	[296]
	CHF rats	Activation of Nrf2/HO-1	[297]
	DOX-induced mice	Inhibition of NOX2 and NOX4 expression	[298]
	Diabetic mice	Regulation of adipocyte factors	[299]
	H9C2 cells	Up-regulation of endogenous antioxidant capacity	[300]
	Diabetic rats	Inhibition of caspase-3 expression and restoration of Bcl-xl expression	[301]
	TAC rats	Activation of SIRT1/NLRP3	[302]
Tanshinone IIA	H9C2 cells	Down-regulation of p38/p53 expression	[303]
	MI rats	Improvement of ER stress and inhibition of cardiomyocyte apoptosis	[304]
*Lycium barbarum* polysaccharide	MI/R rats	Activation of Akt/eNOS	[306]
	HF rats	Down-regulation of cytokine levels and MDA levels	[307]
	H9c2 cells	Up-regulation of endogenous antioxidant capacity and down-regulation of MDA levels	[308]

## Conclusion

As highlighted in this article, maintaining normal physiological levels of ROS is critical for the treatment of OS-related CVDs. First, we briefly review the sources of ROS production, including NOXs, eNOS, ER stress, mitochondrial ETC leakage, and peroxisomes, with eNOS uncoupling leading to impaired protective NO synthesis and increased OS being particularly significant in CVDs. Second, OS-related signaling pathways are equally important for the promotion of PAH, AS and MI. In addition, it is worth noting the interaction of the inflammatory response with OS in the disease process.

Probucol is the only antioxidant drug approved by Food and Drug Administration, and it also shows a very powerful therapeutic effect in CVDs, but it has gradually faded out of the clinical treatment of CVDs because of its side effects such as gastrointestinal discomfort, diarrhea, ventricular tachycardia and severe ventricular arrhythmias [309, 310]. Although antioxidants have not been widely used in the treatment of CVDs to date, they should still be taken seriously, especially natural antioxidants, which remain indispensable as potential therapeutic agents for the treatment of CVDs. Clinical studies have found that natural antioxidants such as RSV, tea polyphenols, BA and quercetin have significant efficacy in the prevention and treatment of CVDs, and have the advantages of low toxicity and fewer adverse effects, which have become a hot spot for research at home and abroad [311–313].

The Chinese herbal monomer antioxidant therapy mentioned in detail in this article can help prevent and treat PAH, AS and MI. For example, herbal monomers have beneficial effects on CVDs by activating SIRT-associated signaling pathways, AMPK-associated signaling pathways, Nrf2-associated signaling pathways, and/or inhibiting the expression of NF-κB-associated signaling pathways and pro-inflammatory factors. Therefore, it is undeniable that herbal monomers are widely used for the prevention and treatment of various diseases due to their remarkable efficacy and high safety. However, herbal monomers targeting OS through their powerful antioxidant therapeutic capacity as a preventive and therapeutic approach to CVDs may provide limited additional benefit. First, because the antioxidant effects of some herbal monomers mentioned in the current paper have only been evaluated in preliminary pharmacological studies, without further research or in-depth study of their molecular mechanisms. Secondly, data on pharmacokinetic and clinical studies of these reported herbal monomers are scarce, and studies on toxicity and its target organs are hardly reported.

Therefore, it is essential that more work should be invested in the future to study the side effects and toxicity of these herbal monomers. Meanwhile, we have little access to data from clinical studies of herbal monotherapy for OS-associated CVDs. Therefore, researchers should be encouraged to further investigate the clinical studies of herbal monomers on CVDs and the side effects and toxicity studies after treatment with herbal monomers to evaluate the actual therapeutic effects of these herbal monomers in humans. In addition, one of the drawbacks of herbal monomers cannot be ignored is their low bioavailability. Perhaps we can consider developing new dosage forms for them to improve their bioavailability or new dosage forms that can reduce their toxicity. In conclusion, the development of safe and effective natural drugs for antioxidant therapy is an important goal for the prevention and treatment of CVDs in the future.

## Data Availability

Not applicable.
